# Impact of Aerobic Exercise on Endothelial Function and Other Cardiovascular Disease Markers in Health and Disease: A Systematic Review and Meta-analysis with Meta-regression of Randomized Controlled Trials

**DOI:** 10.1186/s40798-026-01110-0

**Published:** 2026-10-08

**Authors:** Simon Iskra, Gašper Turnšek, Kristina Drole, Armin Paravlić

**Affiliations:** 1 Faculty of Sport, University of Ljubljana, Gortanova 22, 1000 Ljubljana, Slovenia; 2 Science and Research Centre Koper, Institute for Kinesiology Research, Koper, Slovenia; 3 Faculty of Sport Studies, Masaryk University, 625 00 Brno, Czech Republic

**Keywords:** Physical exercise, Vascular function, Cardiovascular health, Brachial artery flow-mediated dilatation, Biomarkers

## Abstract

**Background:**

Aerobic exercise (AE) has long been recognized as a potent stimulus for improving various cardiovascular disease (CVD)-related markers. Although several meta-analyses have focused on some of these markers, no meta-analysis or meta-regression has comprehensively evaluated the influence of multiple CVD-related markers on endothelial function, assessed by brachial-artery flow-mediated dilation (_ba_FMD).

**Methods:**

This meta-analysis and meta-regression included randomized controlled trials investigating the effect of AE on _ba_FMD. The primary outcome was pre- to post-intervention changes in _ba_FMD. Secondary outcomes included endothelial blood biomarkers, hemodynamic measurements, cardiorespiratory fitness, body composition, glucose metabolism, and lipid profile. Pre- to post-intervention changes were summarized using a randomeffects model. Meta-regression analyses were performed to explore whether changes in secondary outcomes moderated changes in _ba_FMD.

**Results:**

Seventy-two studies comprising 3357 participants were included. AE significantly improved _ba_FMD (mean difference = 2.35%, *p* < 0.001), corresponding to an estimated 24% reduction in CVD risk. Significant improvements were observed across multiple CVD-related markers. Meta-regression revealed that changes in body weight and body fat (*β* = − 4.021 and − 1.721), systolic and diastolic blood pressure (*β* = − 0.525 and − 0.484), maximal oxygen uptake (*β* = 0.656), and insulin levels (*β* = 0.381) significantly moderated _ba_FMD improvements.

**Conclusions:**

AE elicits robust improvements in endothelial function and a wide range of CVD-related markers. This is the first meta-analysis to integrate these outcomes through meta-regression, demonstrating that exercise induced enhancements in body composition, cardiorespiratory fitness, blood pressure and insulin regulation collectively contribute to endothelial adaptations. These findings provide new mechanistic insights into the vascular benefits of AE and reinforce its role as a potent nonpharmacological strategy for CVD prevention and vascular health optimization.

The protocol was prospectively registered in the Open Science Framework online registry (DOI: https://doi.org/10.17605/OSF.IO/E4QYD).

**Graphical Abstract:**

**Figure:**
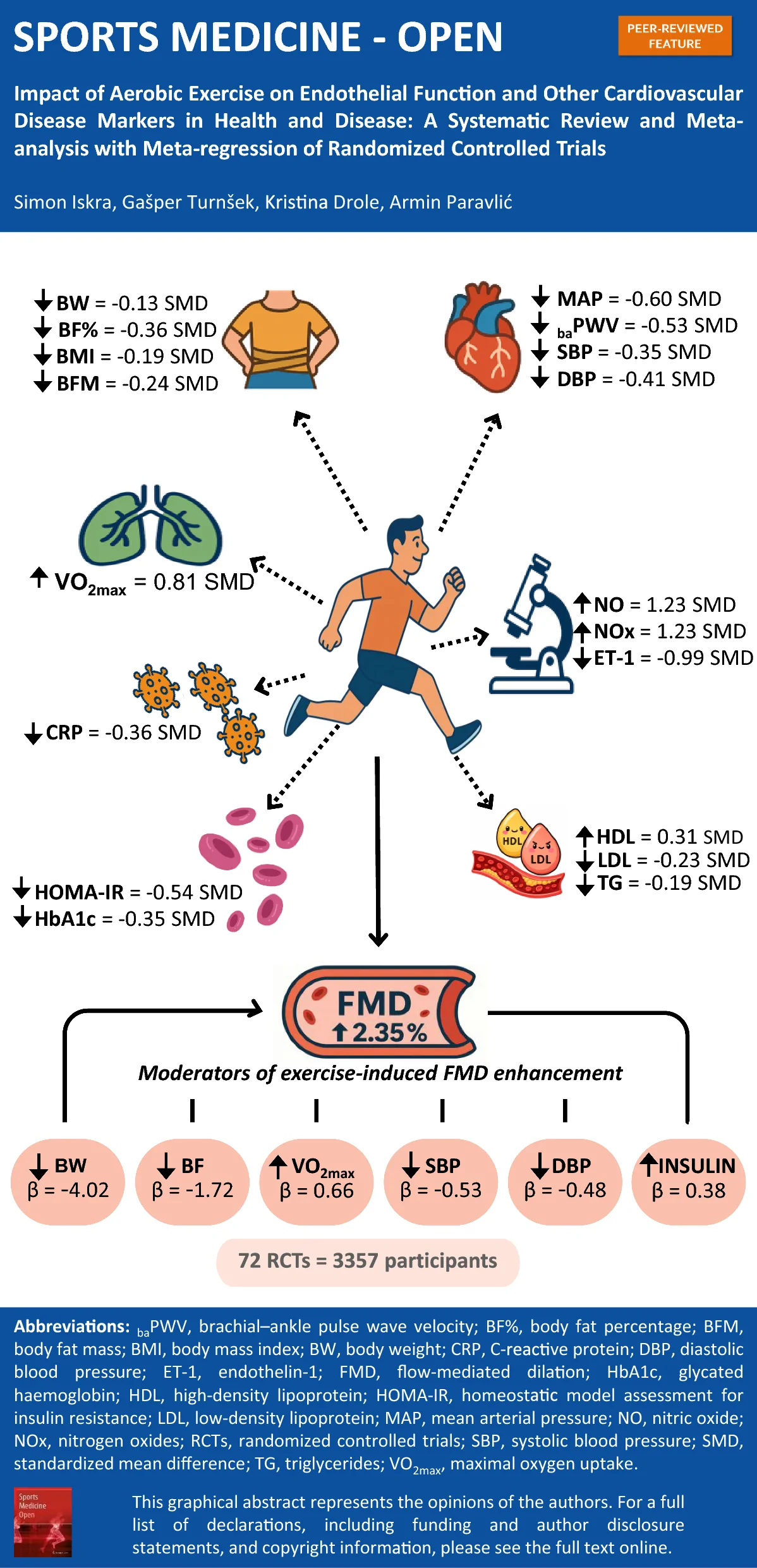

**Supplementary Information:**

The online version contains supplementary material available at https://doi.org/10.1186/s40798-026-01110-0.

## Key Points

- Increases in endothelial function through aerobic exercise are moderated by improvements in body composition, cardiovascular fitness, blood pressure regulation and insulin regulation.
- Aerobic exercise elicits robust improvements across a wide range of physiological functions. These functions work together to mitigate the risk of developing cardiovascular disease.

**Supplementary Information:**

The online version contains supplementary material available at https://doi.org/10.1186/s40798-026-01110-0.

## Introduction

Noncommunicable diseases are one of the major causes of morbidity and mortality, representing a major public health challenge in today's society [1]. In addition to the public health burden, noncommunicable diseases also pose a significant financial burden. Cardiovascular disease (CVD) remains the major cause of mortality and morbidity in Europe, accounting for 45% of annual deaths and one-third of all deaths globally [2]. CVD represents a plethora of different conditions involving dysfunction of the heart, central, and/or peripheral vasculature. The development of CVD is influenced by genetic as well as environmental factors and leads to negative changes in all branches of the cardiovascular system, hormonal homeostasis, dysfunction of other organs, and the immune system [3, 4].

Alterations in the vascular wall primarily impair its functional capacity, as evidenced by increased vascular stiffness, attenuated vasodilatory response to physiological stimuli, and disruptions in multiple interconnected cellular pathways, ultimately leading to endothelial dysfunction. The endothelium is a thin layer of cells lining the interior surface of blood vessels. It is responsible for the regulation of vascular stiffness and vasodilation through the release of various biochemical compounds that regulate vasodilation, vasoconstriction, oxidative processes, cell adhesion, as well as numerous other processes [5, 6]. Therefore, monitoring and improving vascular health are highly important for both primary and secondary CVD prevention, partly because vascular dysfunction is an early marker of the development and progression of CVD.

Flow-mediated dilation (FMD) has emerged as the gold standard for vascular health monitoring through non-invasive high-resolution ultrasound measurements of endothelium-dependent vasodilation after an ischemic stimulus [7]. FMD is commonly used due to its association with coronary artery function and its ability to predict cardiovascular risk [8, 9]. It has been shown that each 1% of absolute increase in brachial artery FMD is associated with an 8 to 13% relative risk decrease in experiencing an adverse cardiovascular event [10], and a stronger association was observed for diseased individuals. Even though FMD is the most commonly used measure to monitor vascular health, the question of how it is associated with other markers of cardiovascular health remains to be answered.

Physical exercise interventions have been recognized as a means of improving vascular health across various populations [11–14]. An umbrella review [15] (number of participants = 5464) has shown that both healthy and diseased populations can expect improvement in FMD specifically following both resistance and aerobic exercise training interventions. While data on the effects of physical exercise on FMD are available in the literature, evidence regarding its impact on other markers of endothelial function is lacking. FMD primarily reflects nitric oxide (NO) bioavailability and vascular regulation. To date, several meta-analyses have addressed this question across various populations. In general, physical exercise is associated with beneficial changes in cardiovascular and metabolic health markers, such as systolic blood pressure, diastolic blood pressure, body fat percentage, insulin, low-density lipoprotein (LDL) cholesterol, high-density lipoprotein (HDL) cholesterol, total cholesterol (tChol), blood glucose, maximal oxygen uptake (VO_2max_) [16–18], as well as circulating blood biomarkers associated with vascular function such as NO, endothelin-1 (ET-1) [19], intracellular adhesion molecule (ICAM), vascular adhesion molecule, and inflammatory biomarkers [16].

Although previous meta-analyses have investigated individual CVD-related biomarkers or endothelial function outcomes in specific populations, the evidence remains fragmented. To date, no meta-analysis or meta-regression has comprehensively evaluated the influence of multiple CVD-related markers on brachial-artery FMD (_ba_FMD) across diverse cohorts. Most available syntheses have restricted their focus to a single marker or to specific cohorts, thereby limiting mechanistic insights into how changes in systemic markers are reflected in endothelial function.

In addition, while individual trials have reported parallel changes in CVD-related markers and _ba_FMD following aerobic exercise (AE), systematic comparison of these associations across studies has not been done. The absence of such synthesis constrains our ability to determine whether changes in circulating markers are mechanistically linked to endothelial adaptations or simply occur in parallel.

A rigorous meta-analysis and meta-regression approach addressing these gaps will provide a more integrated understanding of the relationship between exercise-induced biomarker changes and vascular endothelial function. Such knowledge is crucial for elucidating causal pathways, identifying key mechanistic mediators of _ba_FMD improvements, and ultimately informing the design of more precise physical exercise interventions aimed at optimizing vascular health.

Therefore, the aim of this meta-analysis and meta-regression was to investigate the effects of AE on endothelial function, as measured by _ba_FMD, and on other CVD markers including cardiorespiratory fitness (CRF), body composition, endothelial function, lipid and glucose regulation, and the inflammatory biomarkers. Also, we aimed to investigate the associations of changes from pre- to post- intervention of the aforementioned markers compared to non-exercise control with changes in _ba_FMD via meta-regression analysis.

## Methods

This systematic review and meta-analysis was conducted in accordance with the 2021 PERSiST guidance (implementing Prisma in Exercise, Rehabilitation, Sport medicine and Sport Science) and Preferred reporting Items for Systematic reviews and Meta-analyses (PRISMA) statement. The protocol was prospectively registered in the Open Science Framework online registry (DOI: https://doi.org/10.17605/OSF.IO/E4QYD).

### Search Strategy

We conducted a preliminary systematic search within the scope of a broader project. PubMed, Web of Science, EMBASE, CINAHL, EBSCOhost and the Cochrane Central Register of Controlled trials databases were searched from inception to 15 September 2023. We conducted an additional updated search again on 15 February 2025 and 22 June 2026. Online databases were searched using a combination of medical subject heading terms and search strings: adult, vascular, endothelium, endothelial function, endothelial dysfunction, flow-mediated dilation, FMD, endothelium-dependent vasodilation, vascular reactivity, exercise, physical exercise, training, and randomized controlled trial. Additionally, relevant medical subject headings (MeSH) terms were combined using Boolean operators “OR” and “AND”. Additionally, the Boolean operator “NOT” was included where possible, including words “review” and/or “systematic review” (Supplementary material S1).

### Inclusion and Exclusion Criteria

Eligibility criteria were determined using the Population, Intervention, Comparison, Outcomes, and Study (PICOS) framework. Population: studies recruiting healthy and symptomatic adults (≥ 18 years of age) with no restrictions on sex or ethnicity. Intervention: aerobic training intervention with an eligible non-intervention control group. Comparison: changes in primary and secondary parameters between experimental and control groups. Outcome: we included only trials reporting brachial-artery FMD (_ba_FMD) and at least one of the secondary outcomes (see Table 1).

**Table 1:** Outcomes of interest

Outcomes of interest	
Primary	Brachial-artery flow-mediated dilation (_ba_FMD)
Secondary	Anthropometric measurements: Body weight (BW) Skeletal muscle mass (SMM) Body fat mass (BFM) Percent body fat (BF%) Body mass index (BMI) Hemodynamic measurements: Carotid–femoral pulse wave velocity (_cf_PWV) Brachial–ankle pulse wave velocity (_ba_PWV) Augmentation index (Aix) Mean arterial pressure (MAP) Systolic blood pressure (SBP) Diastolic blood pressure (DBP) Cardiorespiratory measurements: Maximal oxygen uptake (VO_2max_) Gas exchange threshold (GET) Respiratory compensation point (RCP) Respiratory exchange ratio (RER) Blood measurements: High-density lipoprotein (HDL) cholesterol Low-density lipoprotein (LDL) cholesterol Triglycerides (TG) Total cholesterol (tCHOL) Glucose Homeostatic Model Assessment for Insulin Resistance (HOMA-IR) Glycated hemoglobin (HbA1c) C-reactive protein (CRP) Nitric oxide (NO) Nitrogen oxides (NO_x_) Endothelin-1 (ET-1) Insulin Tumor necrosis factor-α (TNF-α) Interleukin 6 (IL-6) Vascular endothelial growth factor (VEGF) Angiotensin Adiponectin

### Selection Process

**Fig. 1:**
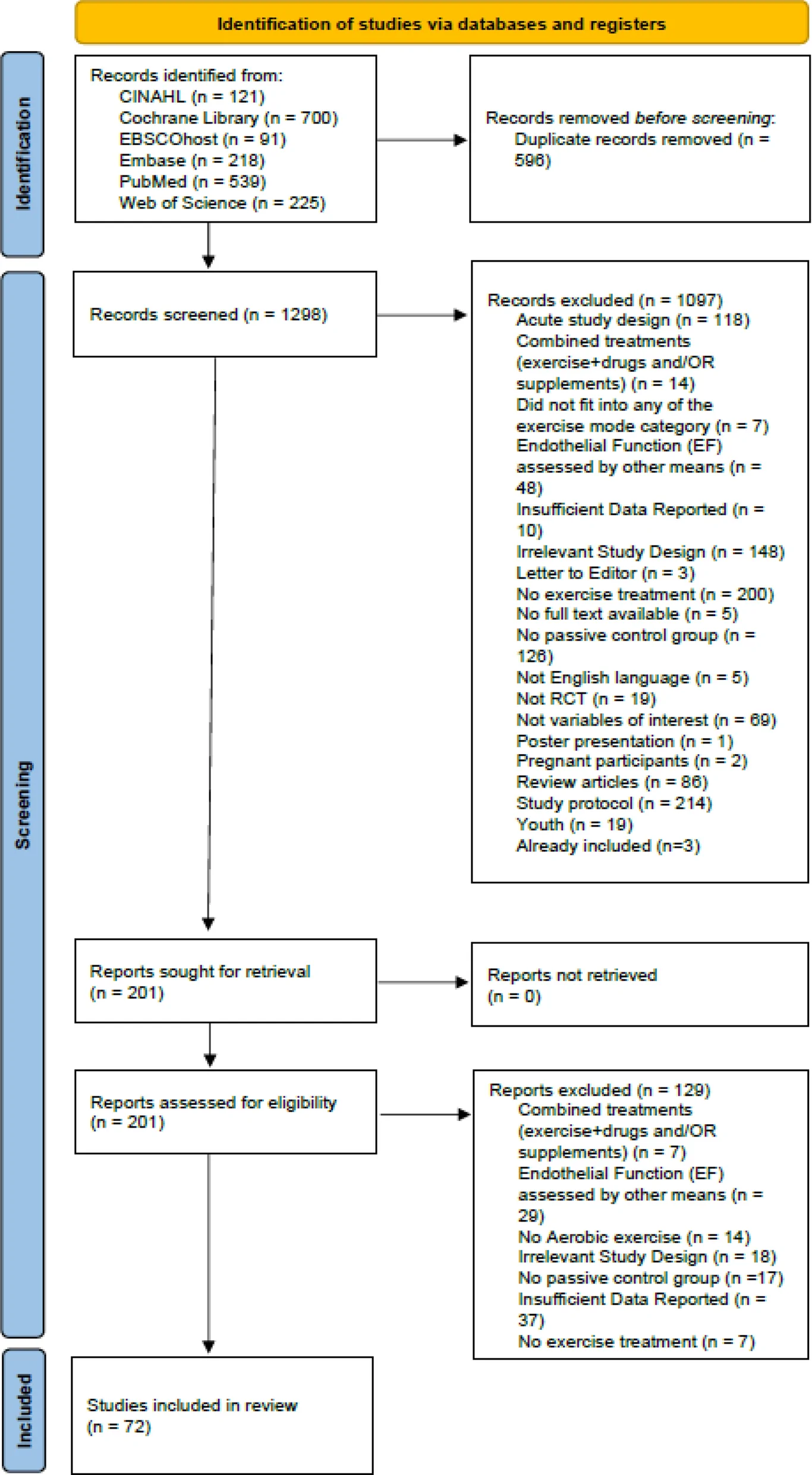
PRISMA study identification and selection flowchart

The literature search and study identification were initially performed by two researchers (AP and NL). Six reviewers (AP, EA, SI, KD, FM, NL) independently assessed the articles for eligibility (see Acknowledgments section for reviewer identification). The full-text articles were reviewed by EA and SI, leading to a final decision on their inclusion. In cases of disagreement, an additional reviewer was consulted (AP), resolving any disagreements by consensus. If the full text was not available online, the corresponding author was contacted by e-mail or through the ResearchGate platform. In the secondary literature search, SI, EA, and NL performed the updated search, whereas SI and GT performed the study identification, including screening the titles and abstracts, as well as the full-text literature search. The study selection process is presented in Fig. 1.

### Data Collection

Data collection was performed by SI and GT. The Cochrane Consumers and Communication Review Group's data extraction protocol was used to extract primary and secondary outcome data, as were the study protocol, study design, and participant information [20]. Means and standard deviations were extracted for the primary outcomes of interest (Table 1). The data presented in graphs only were extracted using Plot Digitizer [21]. Data reported as 95% confidence intervals, interquartile ranges or standard error means were transformed to means and standard deviations where possible, according to the Cochrane Handbook. Participant characteristics (sample size, age, sex, BMI, and clinical status) and study protocol characteristics (intervention type and duration, session duration, weekly frequency, weekly duration, and intensity) were also extracted. Data extraction was checked independently by AP, and any disagreements were resolved by consensus. We used predetermined criteria to stratify participants by age [22] and BMI according to the WHO classification. Intervention intensity stratification was based on the 2020 European Society of Cardiology guidelines [23] (Supplementary Table 2). Additionally, interventions were stratified on the basis of training type (interval or continuous) and intervention duration (1–4 weeks, 5–8 weeks, 9–12 weeks, 12–16 weeks, and >16 weeks).

### Risk-of-Bias Assessment

The Cochrane ROB 2.0 was used for risk-of-bias assessment of selected studies, based on the primary outcome (_ba_FMD). Two researchers (SI and EA) independently assessed the risk of bias for each included study against several criteria: random sequence generation, allocation concealment, blinding of participants, blinding of personnel, and blinding of outcomes. Another reviewer (AP) assessed all risk-of-bias assessments, and disagreement was resolved after consulting with other reviewers (SI and EA). Studies were graded as low, moderate, or high risk of bias. The methodological quality was assessed using the Physiotherapy Evidence Database (PEDro) scale by one reviewer (SI). Methodological quality was graded on a scale from 0–10 as poor (0–3), fair (4–5), good (6–8), and excellent (9–10), based on the criteria proposed by the authors.

### Statistical Analysis

The meta-analyses were conducted using MAS-Meta-Analysis Simplified [24]. The standardized mean differences (SMD) and 95% confidence intervals (CIs) were calculated for the included studies for both experimental and control groups and the pooled effects estimates were calculated as pre- to post-intervention change in the selected outcome compared to non-exercise control. Between-study heterogeneity was assessed using the I^2^ statistics, where values of 25%, 50%, and 75% represented low, moderate, and high statistical heterogeneity, respectively [25]. Additionally, a sensitivity analysis was performed by excluding the study with the largest effect to examine the robustness of the findings. In cases of substantial heterogeneity, a random-effects model was applied to all analyses.

To evaluate the robustness of the findings, a fixed-effects model was additionally calculated and reported for the primary comparisons. Publication bias was assessed by examining the asymmetry of the funnel plot using Egger’s test, and a significant publication bias was considered if the *p*-value was < 0.10. Additionally, a trim-and-fill adjusted analysis was used to determine the robustness of pooled estimate effects [26], using R studio with *metafor* package (v. 2026.06.0). Considering that _ba_FMD is assessed using standardized protocols and is consistently reported in percentage terms, the use of mean difference (MD) provides an appropriate metric for quantitative synthesis. To enhance interpretation beyond statistical significance, the pooled effects were further evaluated using thresholds of minimal clinically important difference (MCID). A 1% absolute increase in _ba_FMD was selected as the clinical threshold [10]. In accordance with this threshold, and in line with previous recommendations [27], _ba_FMD improvements were classified as *trivial* (< 1%), *small* but clinically relevant (> 1% to < 3%), *moderate* (≥ 3% to < 6%), and *large* (≥ 6%).

For secondary outcomes, a standardized mean difference (SMD) was used because the unit of measurement differed and/or methods of assessment varied substantially between studies for the majority of secondary outcomes. SMD was interpreted using the following criteria: trivial (< 0.20), small (0.2–0.60), moderate (0.61–1.20), large (1.21–2.00), very large (2.01–4.00), and extremely large (> 4.00) [28]. Additionally, the pooled estimates for secondary outcomes were also presented as MD, in order to enhance the clinical interpretation.

Furthermore, a meta-regression analysis was conducted to examine whether the effects of exercise on _ba_FMD were moderated by changes in other secondary outcomes, highlighting potential interdependencies in physiological adaptations that may underlie improvements in vascular function. To minimize the risk of overfitting, meta-regression analyses were performed only when at least 10 studies were available for each examined covariate [29]. A significance level of *p* < 0.05 was considered statistically significant.

## Results

### Study Selection Process

The results from the study selection process are outlined in Fig. 1. In brief, 1894 studies were retrieved from the relevant databases. After duplicate removal (*n* = 596), 1298 studies were reviewed for inclusion based on titles and abstracts and 1097 were screened out based on the established PICOS criteria. We reviewed the full text of the remaining 201 studies, screening out 129 studies that did not meet the criteria for inclusion. In total, 72 studies were included in the meta-analysis.

### Participant Characteristics

In total, 72 studies with 99 study arms and a total of 3357 participants (43% females, *M* = 54.2 ± 13.9 years of age, range: 20.5–78 years) were included in the current review. In total, 1951 participants were included in the experimental groups, and 1406 in the control groups. The health status according to the 2021 Global Burden of Disease classification [30] of the participants in the studies was as follows: 17 healthy, and 55 symptomatic (34 cardiovascular diseases, 4 metabolic syndrome, 10 diabetes and kidney diseases, 3 neoplasms, 2 mental disorders, 1 for respiratory and 1 neurological disorders). The participant characteristics are outlined in Table 2. The duration of the included studies ranged from 4–26 weeks. Within the primary and secondary categories, 33% of the study arms were categorized as interval training, and 67% as continuous training. The exercise categorization of the studies is outlined in Supplementary Material (Supplementary Table 1).

**Table 2:** Participants’ baseline characteristics

Study	*N* (Exp/Con)	Sex	% Females	Age	BMI category	GBD classification	∆baFMD experimental group	∆baFMD control group	Direction and magnitude of effect
Hovsepian et al. 2021 [60]	13/13	Female	100	20.5	Obese	Healthy	1.4	− 1.2	↑ Small
Stensvold et al. 2010 [61]	11/11	Both	NR	48.6	Obese	Metabolic syndrome	1.7	0.3	↑ Small
Shenouda et al. 2017^a^ [62]	9/6	Male	0	26.5	Overweight	Healthy	− 0.7	0.4	↓ Small
Shenouda et al. 2017^b^ [62]	10/6	Male	0	27	Overweight	Healthy	− 1.4	0.4	↓ Small
Kwon et al. 2011 [63]	13/15	Female	100	57.2	Overweight	Diabetes and kidney	2.1	− 0.7	↑ Small
Pugh et al. 2014 [64]	13/8	Both	46	47.5	Obese	Metabolic syndrome	3.8	− 0.6	↑ Moderate
Robinson et al. 2016 [65]	10/9	Both	70	31	Obese	Healthy	− 0.9	0.0	↓ Trivial
Almenning et al. 2015 [66]	8/9	Female	100	26.3	Overweight	Healthy	2.0	− 1.2	↑ Moderate
Beck et al. 2013 [67]	13/15	Both	31	20.9	Overweight	Healthy	3.7	− 0.4	↑ Moderate
Boeno et al. 2020 [68]	15/12	Both	47	45.1	Obese	Cardiovascular	1.7	− 0.4	↑ Small
McDermott et al. 2009 [69]	37/28	Both	52	70.1	Obese	Cardiovascular	− 0.2	− 0.7	↓ Trivial
Vona et al. 2009 [70]	52/50	Both	25	57	Overweight	Cardiovascular	5.4	0.8	↑ Moderate
Belardinelli et al. 2006^a^ [71]	15/12	Male	0	54.1	NR	Cardiovascular	2.3	0.0	↑ Small
Belardinelli et al. 2006^b^ [71]	15/10	Male	0	54.1	NR	Cardiovascular	2.4	0.0	↑ Small
Belardinelli et al. 2005 [72]	30/29	Male	0	57	NR	Cardiovascular	2.8	− 0.2	↑ Small
Belardinelli et al. 2008^a^ [73]	44/42	Both	14	58.5	NR	Cardiovascular	2.6	0.2	↑ Small
Belardinelli et al. 2008^b^ [73]	44/42	Both	18	59	NR	Cardiovascular	2.8	0.2	↑ Small
Benda et al. 2015^a^ [74]	10/9	Both	10	65	Overweight	Cardiovascular	− 0.6	0.1	↓ Trivial
Benda et al. 2015^b^ [74]	10/9	Both	0	65.5	Overweight	Cardiovascular	− 0.5	0.1	↓ Trivial
Eleuteri et al. 2013 [75]	10/11	Male	0	64.5	NR	Cardiovascular	1.9	0.5	↑ Small
Guazzi et al. 2004 [76]	16/15	Male	0	53	Overweight	Cardiovascular	3.3	− 0.5	↑ Moderate
Kitzman et al. 2013 [77]	24/30	Both	72	70	Obese	Cardiovascular	− 0.2	− 0.4	↑ Trivial
Kobayashi et al. 2003 [78]	14/14	Both	14	58.5	NR	Cardiovascular	0.2	0.3	↓ Trivial
Sales et al. 2020^a^ [79]	11/8	Both	NR	NR	NR	Cardiovascular	3.5	0.0	↑ Moderate
Sales et al. 2020^b^ [79]	11/8	Both	NR	NR	NR	Cardiovascular	1.8	0.0	↑ Small
Turri-Silva et al. 2021 [80]	5/5	Both	25	58.5	Overweight	Cardiovascular	− 0.4	0.9	↓ Small
Van Craenenbroeck et al. 2010 [81]	21/17	Both	14	62.4	Overweight	Cardiovascular	1.4	− 0.7	↑ Small
Wisløff et al. 2007^a^ [82]	9/9	Both	22	76	Overweight	Cardiovascular	8.1	− 0.4	↑ Large
Wisløff et al. 2007^b^ [82]	9/9	Both	22	75	Overweight	Cardiovascular	4.6	− 0.4	↑ Moderate
Lavrenčič et al. 2000 [83]	14/15	Male	0	52	Obese	Metabolic syndrome	2.0	− 1.2	↑ Moderate
Vona et al. 2004 [84]	28/24	Both	23	56.5	Overweight	Cardiovascular	7.7	2.4	↑ Moderate
Blumenthal et al. 2005 [85]	44/38	Both	28	62.5	Overweight	Cardiovascular	0.3	− 0.8	↑ Small
Westhoff et al. 2007 [86]	24/27	Both	49	68.7	Overweight	Cardiovascular	2.3	0.2	↑ Small
Sixt et al. 2008 [87]	13/10	Both	26	64	Obese	Cardiovascular	3.2	0.2	↑ Moderate
Westhoff et al. 2008 [88]	12/12	Both	54	67.3	Overweight	Cardiovascular	0.3	0.0	↑ Trivial
Desch et al. 2010 [89]	14/12	Both	27	62.3	NR	Cardiovascular	4.4	− 1.1	↑ Moderate
Hermann et al. 2011 [90]	14/13	Both	19	50	Overweight	Cardiovascular	3.1	− 0.3	↑ Moderate
Molmen-Hansen et al. 2012^a^ [91]	23/25	Both	42	52.5	Overweight	Cardiovascular	0.6	0.9	↓Trivial
Molmen-Hansen et al. 2012^b^ [91]	25/25	Both	45	51.9	Overweight	Cardiovascular	4.2	0.9	↑ Moderate
Prakhinkit et al. 2014^a^ [92]	13/13	Female	100	77.9	Overweight	Mental disorders	3.8	− 0.3	↑ Moderate
Prakhinkit et al. 2014^b^ [92]	14/13	Female	100	77.5	Normal	Mental disorders	5.7	− 0.3	↑ Large
Van Craenenbroeck et al. 2015 [93]	19/21	Both	45	53.1	Overweight	Diabetes and kidney	0.6	0.1	↑ Trivial
Sherwood et al. 2016^a^ [94]	51/49	Both	77	51.2	Overweight	Mental disorders	0.9	− 1.5	↑ Small
Sherwood et al. 2016^b^ [94]	53/49	Both	75	52	Obese	Mental disorders	0.5	− 1.5	↑ Small
Azadpour et al. 2017 [95]	12/12	Female	100	57.1	Obese	Healthy	5.2	− 0.5	↑ Moderate
Ghardashi Afousi et al. 2018^a^ [96]	18/17	Both	49	54.5	Overweight	Diabetes and kidney	3.9	0.9	↑ Moderate
Ghardashi Afousi et al. 2018^b^ [96]	17/17	Both	53	53.7	Overweight	Diabetes and kidney	1.0	0.9	↑ Trivial
Berroug et al. 2019 [97]	93/91	Both	39	66.7	Obese	Cardiovascular	− 0.1	− 0.5	↑ Trivial
Boff et al. 2019^a^ [98]	9/9	Both	61	23.5	Normal	Diabetes and kidney	5.5	− 2.6	↑ Large
Boff et al. 2019^b^ [98]	9/9	Both	50	22.3	Normal	Diabetes and kidney	0.2	− 2.6	↑ Small
Bouaziz et al. 2019 [99]	27/29	Both	73	73.6	Overweight	Healthy	0.8	− 0.4	↑ Small
Yoon et al. 2019 [100]	19/18	Both	73	70	Overweight	Cardiovascular	0.5	0.3	↑ Trivial
Isaksen et al. 2019 [101]	19/11	Both	7	67.5	Overweight	Cardiovascular	3.1	− 0.2	↑ Moderate
Kirkman et al. 2019 [102]	16/15	Both	29	58.5	Obese	Diabetes and kidney	1.0	− 1.2	↑ Small
Lee et al. 2019 [103]	15/15	Female	100	46.9	Obese	Neoplasms	1.7	− 5.1	↑ Large
Claes et al. 2020 [104]	60/60	Both	18	60.7	Overweight	Cardiovascular	0.8	− 1.1	↑ Small
Merlo et al. 2020 [105]	10/10	Both	30	70	Overweight	Respiratory	3.0	− 0.4	↑ Moderate
Blumenthal et al. 2021 [106]	52/23	Both	31	65.2	NR	Cardiovascular	0.5	0.2	↑ Trivial
Haynes et al. 2021^a^ [107]	17/16	Both	79	61.9	Overweight	Healthy	2.4	− 0.1	↑ Small
Haynes et al. 2021^b^ [107]	18/16	Both	76	62	Overweight	Healthy	0.5	− 0.1	↑ Trivial
Davoodi et al. 2022^a^ [108]	16/15	Both	42	53.1	Overweight	Diabetes and kidney	3.0	0.9	↑ Small
Davoodi et al. 2022^b^ [108]	16/15	Both	45	54.3	Overweight	Diabetes and kidney	2.6	0.9	↑ Small
Collins et al. 2023^a^ [109]	15/14	Male	0	51.2	Overweight	Healthy	3.4	0.8	↑ Small
Collins et al. 2023^b^ [109]	15/14	Male	0	50.2	Overweight	Healthy	4.0	0.8	↑ Moderate
Collins et al. 2023^c^ [109]	15/14	Male	0	49.3	Overweight	Healthy	5.0	0.8	↑ Moderate
Hansen et al. 2023 [110]	8/7	Both	47	50	Overweight	Neurological	0.1	− 1.8	↑ Small
Twerenbold et al. 2023 [111]	19/19	Both	37	57.5	Overweight	Cardiovascular	0.1	0.0	↑ Trivial
Braith et al. 2008 [112]	9/7	Both	19	54.4	Overweight	Cardiovascular	− 0.4	− 2.6	↑ Small
Jones et al. 2014 [113]	25/25	Male	0	59.5	Overweight	Neoplasms	1.7	0.3	↑ Small
Belardinelli et al. 2008 [114]	30/26	Both	16	59	Overweight	Cardiovascular	3.2	0.0	↑ Moderate
Tjønna et al. 2008^a^ [31]	8/9	Both	47	50.8	Obese	Metabolic syndrome	4.7	− 0.8	↑ Moderate
Tjønna et al. 2008^b^ [31]	11/9	Both	55	52.5	Obese	Metabolic syndrome	9.0	− 0.8	↑ Large
Yoshizawa et al. 2010 [115]	10/10	Female	100	57.5	Normal	Healthy	1.1	− 0.2	↑ Small
Pierce et al. 2011 [116]	26/10	Both	58	61.5	Overweight	Healthy	1.2	0.2	↑ Small
Abdi et al. 2021 [117]	15/15	Female	100	NR	Overweight	Diabetes and kidney	6.4	0.1	↑ Large
He et al. 2022^a^ [118]	15/15	Female	100	58	Normal	Healthy	0.3	− 0.1	↑ Trivial
He et al. 2022^b^ [118]	8/15	Female	100	41.3	Normal	Healthy	1.1	− 0.1	↑ Small
He et al. 2022^c^ [118]	10/15	Female	100	57.1	Normal	Healthy	3.2	− 0.1	↑ Moderate
He et al. 2022^d^ [118]	15/15	Female	100	58	Normal	Healthy	0.6	0.1	↑ Trivial
He et al. 2022^e^ [118]	8/15	Female	100	41.3	Normal	Healthy	2.1	0.1	↑ Small
He et al. 2022^f^ [118]	10/15	Female	100	57.1	Normal	Healthy	4.6	0.1	↑ Moderate
He et al. 2022^g^ [118]	15/15	Female	100	58	Normal	Healthy	1.1	0.1	↑ Trivial
Hwang et al. 2019^a^ [119]	18/15	Both	NR	NR	NR	Diabetes and kidney	0.9	− 0.3	↑ Small
Hwang et al. 2019^b^ [119]	16/15	Both	NR	NR	NR	Diabetes and kidney	0.9	− 0.3	↑ Small
Suboc et al. 2014^a^ [120]	36/41	Both	31	63	Overweight	Healthy	0.2	0.7	↓ Trivial
Suboc et al. 2014^b^ [120]	30/41	Both	31	62.5	Overweight	Healthy	3.3	0.7	↑ Small
Pereira et al. 2015 [121]	30/30	Both	NR	NR	NR	Healthy	4.1	− 0.4	↑ Moderate
Ploydang et al. 2023 [122]	16/17	Both	64	69.1	Normal	Diabetes and kidney	2.4	− 0.3	↑ Small
Isaksen et al. 2014 [123]	24/11	both	9	66.3	Overweight	Cardiovascular	3.5	− 0.2	↑ Moderate
Eaungpulsawat et al. 2023 [124]	12/12	Female	100	68.1	Overweight	Cardiovascular	2.3	− 0.9	↑ Moderate
Siripanya et al. 2023 [125]	11/11	Female	100	45	Normal	Neoplasms	2.2	0.0	↑ Small
Oliveira e Silva et al. 2019 [126]	14/14	Both	46	54	Overweight	Diabetes and kidney	2.2	− 2.0	↑ Moderate
Aispuru-Lanche et al. 2024^a^ [127]	28/24	Both	17	58	Overweight	Cardiovascular	2.6	0.5	↑ Small
Aispuru-Lanche et al. 2024^b^ [127]	28/24	Both	19	57.9	Overweight	Cardiovascular	5.6	0.5	↑ Moderate
Goeder et al. 2024 [128]	23/12	Male	NR	51.1	NR	Healthy	2.1	− 0.6	↑ Small
Huang et al. 2025^a^ [129]	16/16	Both	NR	21.8	Normal	Healthy	3.1	− 0.2	↑ Moderate
Huang et al. 2025^b^ [129]	15/16	Both	NR	22	Normal	Healthy	4.3	− 0.2	↑ Moderate
Yu et al. 2025^a^ [130]	21/15	Both	24	23.2	Overweight	Healthy	2.7	− 1.6	↑ Moderate
Yu et al. 2025^b^ [130]	21/15	Both	27	24.3	Overweight	Healthy	1.9	− 1.6	↑ Moderate

^a,b,c,d,e,f,g^Represent different study arms within one study; *N*: number of participants; Exp: experimental; Con: control; BMI: body mass index; GBD: global burden of disease; ∆baFMD: pre-to-post intervention change in brachial artery flow-mediated dilation; NR: not reported

### Methodological Quality

**Fig. 2:**
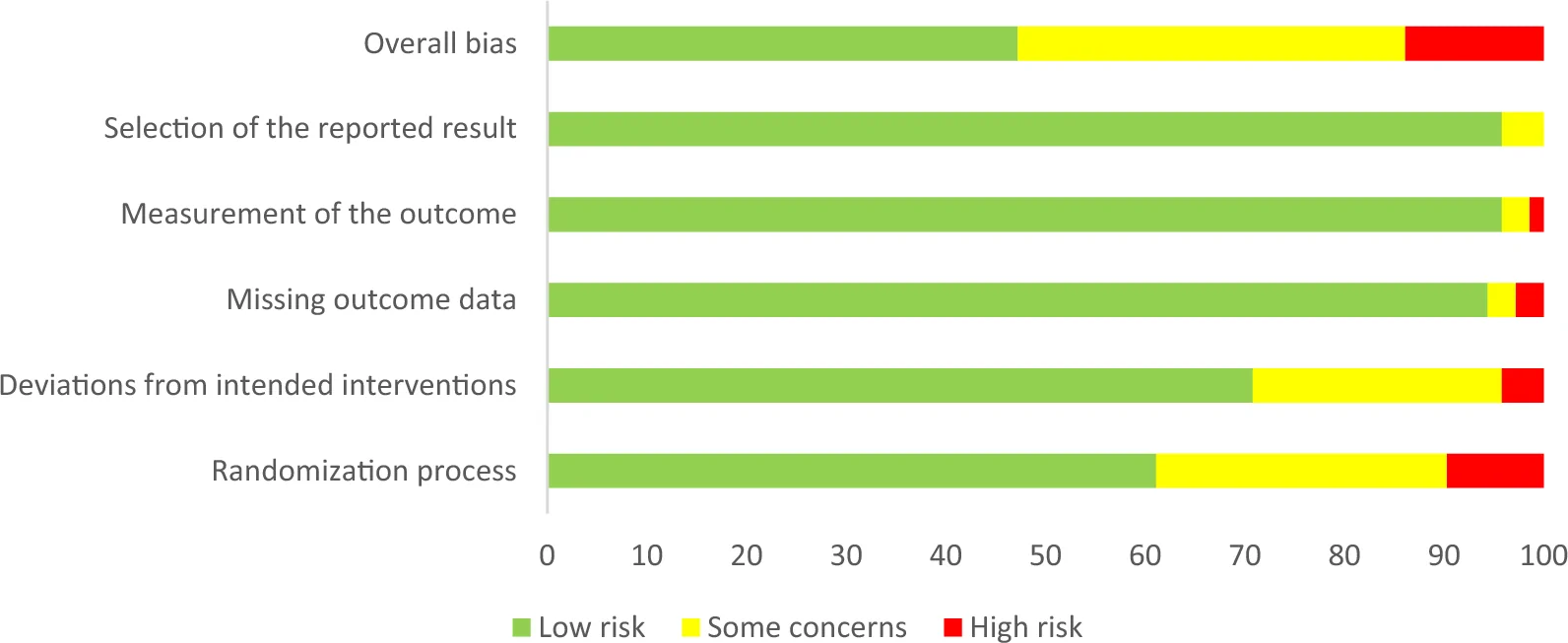
Risk-of-bias assessment (intention-to-treat), expressed as percentage

The methodological quality of the included studies was on average good, with an average PEDro score of 5.6 (median = 6, range from 3 to 8). Based on predetermined thresholds, 3%, 44% and 53% of studies were defined as having poor, fair, and good quality, respectively. The risk-of-bias assessment results revealed that 47% of studies had a low risk of bias, 39% had some concerns, and 14% had a high risk of bias. The main risk of bias stemmed from concerns regarding the randomization process (39%), deviations from intended interventions (29%), missing outcome data (6%), measurement of the outcome (4%), and selection of the reported result (4%). We acknowledge that the blinding of participants and therapists in exercise intervention trials is challenging. Detailed information about the study quality and risk of bias is presented in Fig. 2 and Supplementary Material (Supplementary Table 2).

#### Publication Bias Assessment

The results of Egger’s test indicated publication bias for the meta-analysis summarizing results for VO_2max_ (*p* = 0.014), Aix (*p* = 0.035), SBP (*p* = 0.021), BF% (*p* = 0.08), and HOMA-IR (*p* = 0.02) only, whereas meta-analyses for RER (*p* = 0.47), GET (*p* = 0.57), BW (*p* = 0.21), BFM (*p* = 0.17), SMM (*p* = 0.44), _cf_PWV (*p* = 0.72), MAP (*p* = 0.43), _ba_PWV (*p* = 0.85), DBP (*p* = 0.11), HDL cholesterol (*p* = 0.78), LDL cholesterol (*p* = 0.24), TG (*p* = 0.2), tCHO (*p* = 0.31), HbA1c (*p* = 0.99), CRP (*p* = 0.15), NO (*p* = 0.19), nitrogen oxide (NO_x_) (*p* = 0.75), ET-1 (*p* = 0.26), insulin (*p* = 0.87), TNF-α (*p* = 0.11) and IL-6 (*p* = 0.9) were unbiased.

### Effects of Aerobic Exercise Intervention on Brachial Artery Flow-Mediated Dilatation

**Fig. 3:**
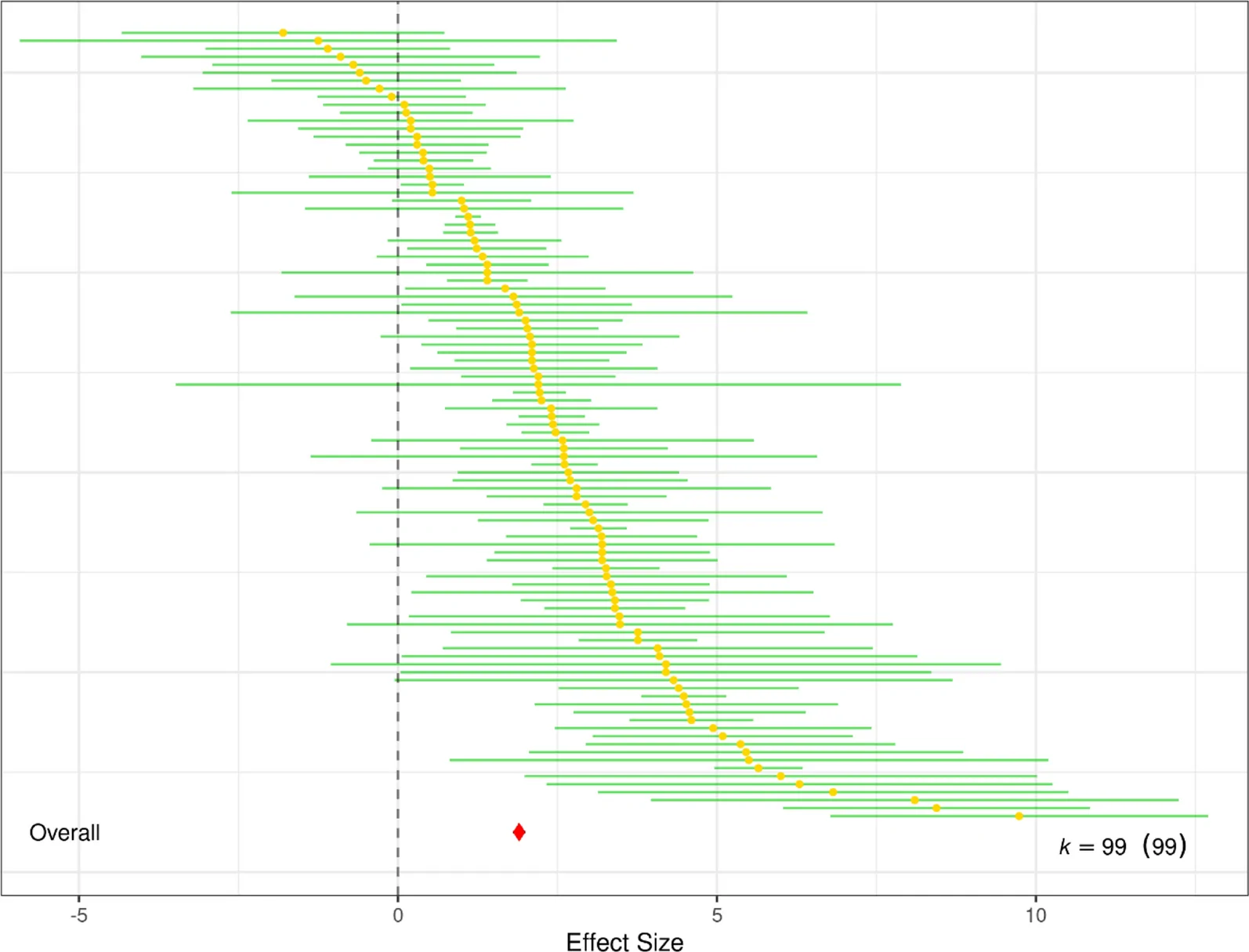
Changes in _ba_FMD after aerobic exercise interventions compared to non-exercise control. Yellow ○ represent mean difference with 95% confidence intervals for each experimental group compared to non-exercise control. The pooled mean difference is represented with red ◊, along with 95% confidence intervals; *k*: number of comparisons

Meta-analysis of 99 effect sizes demonstrated a *small* but significant improvement of _ba_FMD following the aerobic exercise intervention compared to non-exercise control (Fig. 3). The overall MD, estimated using a random-effects model, was 2.35% (95% CI 1.98 to 2.73; *p* < 0.0001), which corresponds to a small but clinically meaningful effect. Consistent results were obtained using a fixed effects model, yielding a mean difference of 1.90% (95% CI 1.80 to 2.00; *p* < 0.0001), thereby supporting the robustness of the effect across different analytic approaches. Following trim-and-fill adjustment, the pooled estimate was attenuated but remained statistically significant (adjusted MD = 1.74%; 95% CI 1.31 to 2.18; *p* < 0.0001). Substantial heterogeneity was observed in the primary random-effects model (I^2^ = 90.40%; *p* < 0.0001) and persisted after trim-and-fill adjustment (I^2^ = 93.90%; *p* < 0.0001), indicating considerable between-study variability.

**Fig. 4:**
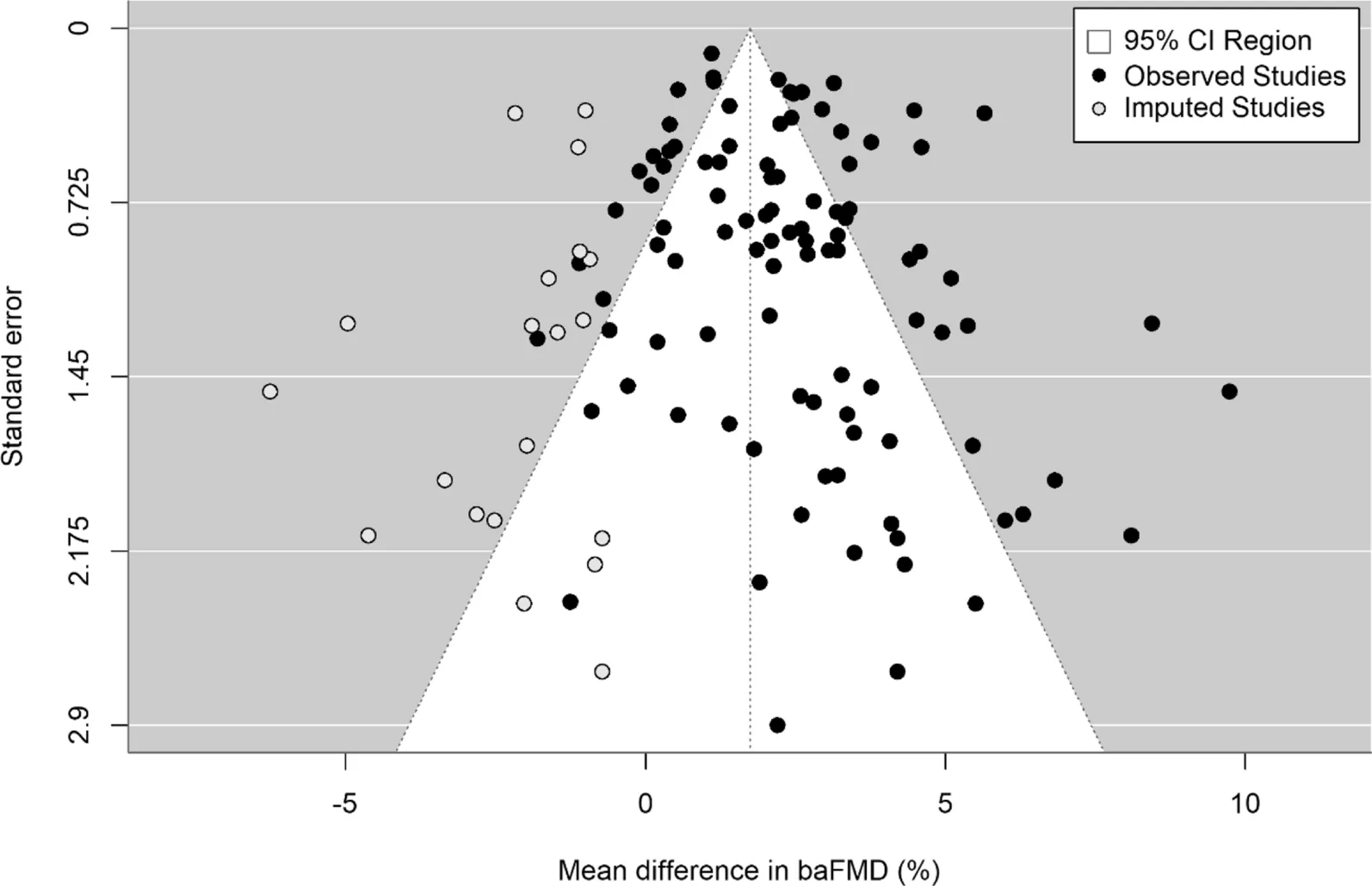
Funnel plot of mean differences vs. standard errors for _ba_FMD, including trim-and-fill adjusted estimates. CI: confidence intervals; baFMD: brachial artery flow-mediated dilation

Egger’s regression test revealed evidence of potential publication bias in the overall meta-analysis of _ba_FMD (*p* = 0.0102). The funnel plot also demonstrated visible asymmetry. To examine the potential influence of small-study effects, a trim-and-fill adjusted analysis was performed (Fig. 4). The adjusted pooled estimate remained statistically significant and was broadly consistent with the primary analysis. Additionally, to assess the robustness of the findings, a sensitivity analysis was conducted by removing the study with the largest MD [31]. The overall summary estimate remained statistically significant. However, publication bias persisted (*p* = 0.0129).

The magnitude and direction of effects, and the associated 95% CIs were calculated for the pooled summary estimates. The results are presented in Supplementary Material (S6).

### Meta-regression Analysis

Table 3 shows the results of the meta-regression, which examined whether pre- to post-intervention changes in anthropometric, hemodynamic, hematological parameters, and CRF moderated the effects of aerobic training on _ba_FMD. The analyses revealed that several variables significantly influenced the effects of aerobic training on endothelial function. A significant negative association was found for changes in body weight (*β* = − 4.021; *p* = 0.008), indicating that greater reductions in body weight were associated with more pronounced improvements in _ba_FMD. Similarly, changes in body fat also significantly moderated the outcome (*β* = − 1.721; *p* = 0.004), supporting the role of fat loss as a contributor to vascular adaptations. Among hemodynamic parameters, both systolic (*β* = − 0.525; *p* = 0.007) and diastolic blood pressure (*β* = − 0.484; *p* = 0.001) showed significant negative associations, suggesting that greater reductions in blood pressure after a period of aerobic training may enhance endothelial function. In addition, changes in insulin levels significantly moderated the training effect (*β* = 0.381; *p* = 0.034), while improvements in VO_2__max_ also showed a strong positive association with changes in _ba_FMD (*β* = 0.656; *p* = 0.001). No significant moderating effects were observed for other variables, including BMI, MAP, HDL, LDL, triglycerides, total cholesterol, glucose, HOMA-IR, and HbA1c.

**Table 3:** Meta-regression analysis for predictors of effects of aerobic exercise training on _ba_FMD

Parameters of interest	*β* coefficient	Standard error	*t* value	*p* value	95% lower CI	95% upper CI	Df
Changes from pre-to-post intervention							
Body weight (SMD)	− 4.021	1.517	− 2.650	0.008*	− 6.995	− 1.047	36
Body mass index (SMD)	− 0.665	0.420	− 1.585	0.112	− 1.488	0.157	47
Body fat (SMD)	− 1.721	0.595	− 2.890	0.004*	− 2.888	− 0.554	17
Systolic blood pressure (mm/Hg)	− 0.525	0.196	− 2.680	0.007*	− 0.908	− 0.141	53
Diastolic blood pressure (mm/Hg)	− 0.484	0.151	− 3.210	0.001*	− 0.780	− 0.188	51
Mean arterial pressure (SMD)	− 0.746	0.786	− 0.948	0.343	− 2.286	0.795	8
High-density lipoprotein cholesterol (SMD)	− 0.041	0.164	− 0.250	0.802	− 0.363	0.281	41
Low-density lipoprotein cholesterol (SMD)	− 0.410	0.350	− 1.171	0.242	− 1.097	0.276	37
Triglycerides (SMD)	− 0.254	0.286	− 0.888	0.374	− 0.815	0.306	38
Total cholesterol (SMD)	− 0.149	0.172	− 0.866	0.386	− 0.487	0.189	38
Glucose (SMD)	− 0.253	0.377	− 0.670	0.503	− 0.992	0.486	26
HOMA-IR (SMD)	− 0.258	0.305	− 0.846	0.398	− 0.856	0.340	14
HbA1c (SMD)	0.290	0.643	0.451	0.652	− 0.970	1.551	9
Insulin (SMD)	0.381	0.179	2.125	0.034*	0.030	0.732	14
VO_2max_ (SMD)	0.656	0.180	3.646	0.001*	0.303	1.008	52

HOMA-IR: homeostatic model assessment for insulin resistance; HbA1c: glycated hemoglobin; VO_2max_: maximal oxygen uptake; CI: confidence interval; SMD: standardized mean difference; Df: degrees of freedom; * statistically significant at p < 0.05

## Discussion

This systematic review and meta-analysis aimed to investigate the effects of AE on a comprehensive panel of endothelial, CRF, anthropometric, and various hemodynamic and biochemical markers associated with CVD risk. The primary aim of this study was to appraise the existing RCTs, primarily examining the effect of AE on endothelial function, assessed by _ba_FMD. Additionally, we aimed to examine the association between changes in other independent markers of CV health and _ba_FMD following AE intervention. In total, 72 RCTs, consisting of 99 study arms with 3357 participants, were included in the meta-analysis.

We found that AE exhibits a positive and clinically meaningful effect on _ba_FMD (2.35% [95% CI 1.98 to 2.73; *p* < 0.001]) compared to non-exercise control, indicating an approximately 24% decreased risk of future cardiovascular events [32]. The secondary findings revealed that AE exhibits a significant effect on body weight decrease, and markers of endothelial function (NO and NO_x_), with small-to-moderate improvements in blood pressure reduction, arterial stiffness, VO_2max_, glucose regulation, lipid profile, and CRP. Our findings support the notion of cardioprotective effects of AE on several different structural and functional mechanisms involved in CV health regulation [33]. The results of the current meta-regression analysis provide evidence for the modulation of _ba_FMD improvement by body composition, CRF, blood pressure, and glucose regulation.

To the best of the authors' knowledge, this is the first systematic review and meta-analysis to examine the effects of AE on _ba_FMD and other structural and functional clinical end-points associated with CV health in adults.

### Vascular Adaptations

Several systematic reviews and meta-analyses have found physical exercise interventions (PEI) as beneficial for improving endothelial function in healthy [15], hypertensive [19], overweight/obese, and Type 2 diabetes affected populations [18]. During AE, shear stress increases significantly as a result of increased blood flow to the working muscles. This results in a strong vasodilatory response in the conduit vessels and provides a potent stimulus for improvements in vasodilatory capacity [34]. Other exercise modalities may also improve endothelial function due to activation of vasoregulatory mechanisms and cellular pathways connected to endothelial function. Yet, recent reviews [15, 35] revealed that AE was the superior exercise modality as opposed to resistance exercise. When examining the effect of AE on endothelial function, exercise intensity and mode of exercise should also be taken into account. Several meta-analyses [18, 35, 36] have examined the effect of moderate-intensity and high-intensity interval exercise on endothelial function. Their results show greater effects on endothelial function with high-intensity interval training compared to moderate-intensity continuous training. This can be attributed to the ability of interval exercise to induce greater transient increases in shear stress during intervals of high intensity compared to smaller continuous increases in shear stress during moderate-intensity exercise. Irrespective of exercise intensity and modality, the results of this meta-analysis revealed a significant improvement in _ba_FMD following AE. On the basis of existing evidence, a 1% improvement in _ba_FMD translates to a 10% attenuated CVD risk [32]. Following this methodology, the 2.35% improvement in _ba_FMD presented in this study translates to a ~23.5% CVD risk reduction. Overall, our findings show promising results regarding the effectiveness of PEI as a nonpharmacological intervention for _ba_FMD improvement, which is in line with the existing literature.

Shear stress is thought to be the main driver of endothelial adaptation following PEI, specifically the activation of endothelial NO synthase and consequently increased NO release [34, 37, 38]. This leads to a change in the regulation of various compounds involved in vasodilation and vasoconstriction response. Our findings confirm this hypothesis as we observed a large increase in NO (SMD = 1.23, *p* < 0.001) and NO_x_ (SMD = 1.23, *p* < 0.001) and a moderate decrease in ET-1 following AE (SMD = − 0.99, *p* < 0.001) (Supplementary Material S6, Figs. 7–9). The observed improvements in NO, NO_x_, and reductions in ET-1 provide critical insights into the mechanisms underlying improved _ba_FMD. NO and ET-1 are primary modulators of endothelial function, and directly influence vascular tone through their vasodilatory and vasoconstrictive actions, respectively. Enhanced NO bioavailability and reduced ET-1 levels following aerobic exercise training likely contribute to improved endothelium-dependent vasodilation, thereby explaining the observed improvements in _ba_FMD. These results validate previously proposed mechanisms [9, 39]. Although our meta-regression did not identify a statistically significant modulatory effect of NO, NO_x_, and ET-1 specifically on _ba_FMD, this is likely attributable to the limited number of studies and inherent variability associated with biomarker measurements. Nonetheless, the clear directional changes observed strongly support the physiological relevance of these biomarkers in enhancing endothelial function. These findings have significant clinical implications. Targeting endothelial dysfunction through lifestyle or pharmacological interventions aimed at improving these biomarkers could present a viable therapeutic strategy for hypertension management and cardiovascular risk reduction. In the future, original research studies should aim to recruit a greater number of participants to further elucidate the precise modulatory roles of NO, NO_x_, and ET-1 in _ba_FMD improvements following AE and their collective impact on cardiovascular health.

### Hemodynamics

In addition to shear stress, numerous other molecular pathways are activated during AE. These changes result in functional and structural changes in the endothelium and vessel wall, resulting in increased luminal diameter, greater elastic content in the vessel wall, and decreased artery wall thickness [38, 40, 41]. These changes also affect arterial wall stiffness, which is prominently measured with PWV and Aix. Our results show a small decrease in peripheral arterial stiffness (_ba_PWV) following AE (SMD = − 0.53, *p* = 0.03), suggesting beneficial effects of AE (Supplementary material S6, Fig. 2). Interestingly, we did not observe central arterial stiffness adaptations following AE, when measured using _cf_PWV and Aix. This might be attributed to the fact that only studies measuring _ba_FMD were included in this meta-analysis, and studies using _cf_PWV as a primary outcome were not included in the analysis. Only 13 independent comparisons from 9 studies were included for _cf_PWV, and 9 independent comparisons from 7 studies were included in the analysis of AIx. Studies examining both _ba_FMD and PWV concurrently are rare, due to methodological complexity, and research focusing mostly on _ba_FMD. Our results regarding PWV and AIx contradict the established evidence, probably due to the small number of included studies [42]. Several studies reported concurrent improvements in FMD and PWV (i.e., increased FMD and reduced PWV) after exercise intervention [43], and, therefore, an effect of AE on PWV should be expected. We found that peripheral measures of arterial stiffness (i.e., _ba_PWV) exhibited a small significant decrease following AE, whereas central measures (i.e., _cf_PWV) did not follow this pattern. This result might be explained by the fact that the time course of vascular stiffness adaptations favors peripheral adaptations rather than central adaptations [44]. Improvements in central measures of arterial stiffness may be expected after at least 10 weeks, whereas peripheral arterial stiffness adaptations may occur earlier [45], as peripheral arteries exhibit enhanced intrinsic plasticity compared to the central arteries (i.e., carotids) [46]. The duration of studies examining PWV ranged from 8–16 weeks (median = 12). Although the median duration of the included studies aligns with the time frame generally required to observe changes in PWV, the limited number of comparisons may have reduced the statistical power needed to detect meaningful changes. Furthermore, the heterogeneity in exercise modalities, intensity, and total training volume applied across studies likely contributed to the variability in outcomes. These factors may have masked potential improvements in _cf_PWV, despite sufficient intervention duration.

The positive functional and structural adaptations following PEI result in improved hemodynamic regulation (i.e., SBP and DBP). Our results corroborate a positive effect of AE on SBP and DBP, with small significant decreases in SBP (SMD = − 0.35, *p* < 0.001), DBP (SMD = − 0.41, *p* < 0.001), and MAP (SMD = − 0.60, *p* = 0.0029) following AE (Supplementary Material S6, Figs. 4–6). SBP and DBP tend to decrease after AE due to an improvement in vascular tone regulation. Notably, the present literature yields heterogeneous results regarding the magnitude of blood pressure reduction [47]. While a recent network meta-analysis [48] found different types of exercise are effective for SBP and DBP reductions, another meta-analysis [49] reported that resistance exercise is more effective for reducing BP in normotensive individuals, while aerobic exercise is more effective for reducing BP in hypertensive individuals. Other reviews [18, 50] highlighted improvements in both SBP and DBP after moderate and high-intensity exercise interventions. We found improvements in SBP and DBP, as expected, considering the present body of knowledge. Our results clearly indicated a significant negative interaction effect between improvements in _ba_FMD and both SBP (*β* = − 0.525, *p* = 0.007) and DBP (*β* = − 0.484, *p* = 0.001), suggesting that improvements in _ba_FMD moderated improvements in SBP and DBP. The observed negative association between improved _ba_FMD and reductions in both SBP and DBP highlights endothelial function as a potential mechanistic mediator of blood pressure regulation. Improvements in endothelial function, as reflected by increased _ba_FMD, suggest an enhanced vasodilatory capacity, leading to decreased vascular resistance and subsequent reductions in both systolic and diastolic pressures. These findings reaffirm the critical role of endothelial health in blood pressure regulation and highlight endothelial function enhancement as a key therapeutic target for reducing cardiovascular risk related to elevated blood pressure.

### Cardiorespiratory Fitness

Improvements in CRF following AE are expected, as AE is a potent stimulus for improvement in key regulatory mechanisms connected with CRF. We focused on ventilatory parameters obtained during a cardiorespiratory exercise test to ensure reliable results. Consequently, data examining CRF through indirect means or other tests were not included in this analysis. The pooled effects of the included studies revealed a moderate increase in VO_2max_ following AE (SMD = 0.81, *p* < 0.001). Furthermore, meta-regression analysis revealed a strong positive association between changes in VO_2max_ and _ba_FMD (*β* = 0.656, *p* = 0.001), suggesting that greater improvements in CRF result in greater improvements in endothelial function.

Importantly, this relationship is likely bidirectional rather than purely unidirectional. While improvements in CRF may enhance endothelial function through repeated increases in shear stress, increased NO bioavailability, and improved vascular homeostasis, preserved endothelial function may also facilitate improvements in CRF by optimizing skeletal muscle perfusion, oxygen delivery, and exercise-induced vascular adaptations. Supporting this concept, McLellan et al. [51] reported that AE elicited similar improvements in VO_2max_ in individuals with preserved and impaired endothelial function, suggesting that aerobic training remains effective across different levels of vascular health. Conversely, Königstein et al. [52] recently reported that lower baseline _ba_FMD may attenuate improvements in VO_2__peak_ following aerobic training, proposing that pre-existing endothelial dysfunction could reduce exercise trainability through impaired endothelial NO synthase signaling and reduced mitochondrial biogenesis. Interestingly, these findings can be interpreted alongside our recently published network meta-analysis, in which higher baseline _ba_FMD was associated with greater improvements in endothelial function following exercise. Collectively, these findings suggest that preserved endothelial function may facilitate vascular adaptations to exercise while simultaneously supporting improvements in CRF. Nevertheless, because our meta-regression is based on study-level associations, the direction of causality cannot be established, and the observed relationship should therefore be interpreted as reflecting interconnected physiological adaptations rather than a simple cause-and-effect pathway.

These observations are biologically feasible because improvements in CRF arise from coordinated functional and structural adaptations involving the working musculature, conduit vessels, and cardiorespiratory system [53]. In addition to enhancing pathways not directly related to endothelial function, such as cardiac contractility and mitochondrial biogenesis, aerobic exercise also modulates molecular pathways that directly influence endothelial health [54]. These adaptations include increased vasodilatory regulation, enhanced capillary density, improved muscular oxidative capacity, and attenuation of chronic inflammation [7]. Consequently, several molecular pathways are likely to contribute simultaneously to improvements in both CRF and endothelial adaptations.

One such shared mechanism may be the attenuation of systemic inflammation. Consistent with this concept, our meta-analysis demonstrated a small reduction in CRP following AE. TNF-α showed a moderate decrease that did not reach statistical-significance using the random-effects model, whereas a significant reduction was observed under the fixed-effects model. Numerous inflammatory biomarkers have been investigated in the literature; however, only CRP, TNF-α and IL-6 could be synthesized in the present analysis because of the limited number of studies reporting other inflammatory markers, consistent with the existing literature (Supplementary material S6, Figs. 26–28).

### Body Composition

AE led to significant reductions in body weight and fat mass. Our results corroborate the existing literature, showing a trivial decrease in BW (SMD = − 0.130, *p* = 0.024), small decrease in BF% (SMD = − 0.36, *p* = 0.006) and a trivial decrease in BMI (SMD = − 0.19, *p* < 0.001) (Supplementary Material S6, Figs. 13–16). Because this meta-analysis applied strict inclusion criteria and focused solely on studies examining the effects of AE in isolation, studies that combined AE with dietary interventions were excluded. Nevertheless, our results demonstrate that AE interventions alone are effective in improving body composition markers, even in the absence of dietary modifications. Although our results show improvements in body fat composition measurements, no effect on skeletal muscle mass was observed. While AE interventions may be effective for improving body fat composition, resistance or combined exercise may be necessary for concomitant increases in muscle mass. The meta-regression results indicated a moderate negative association between improvements in body weight (*β* = − 4.02, *p* = 0.008), body fat (*β* = − 1.721, *p* = 0.004) and _ba_FMD, indicating that decreases in body fat result in greater improvements in endothelial function. Taken together, the aforementioned reduced chronic inflammation might be the result of reducing body mass and, in turn, increasing endothelial function, as inflammation is tightly linked to angiogenesis and consequently endothelial function [55, 56].

### Lipid Profile, Glucose Metabolism and Inflammation Markers

Thus far, we have shown that AE is a potent stimulus for vascular adaptations, CRF and body composition, but it is also effective for improving lipid and glucose metabolism, as well as inflammation. Owing to increased energy demand during AE, glucose and fat metabolism are significantly increased. Chronic adaptations lead to improvement in blood lipid profile by regulating lipoprotein lipase expression and activity, resulting in increased HDL cholesterol and decreased TG levels [57]. Our results are in agreement with the proposed mechanisms and results from previous studies and reviews [16, 58]. We detected a small increase in HDL cholesterol (SMD = 0.31, *p* = 0.003) and a small decrease in TG (SMD = − 0.19, *p* = 0.003) and LDL cholesterol (SMD = − 0.23, *p* < 0.001) (Supplementary Material S6, Figs. 18–20). In contrast, the results from meta-regression did not exhibit significant moderating effects of blood lipid profile markers on _ba_FMD, suggesting these changes are mainly regulated by other mechanisms than those regulating endothelial function.

In addition to the blood lipid profile, our results indicate a small decrease in fasting glucose (SMD = − 0.27, *p* < 0.001), HbA1c (SMD = − 0.35, *p* = 0.015), and HOMA-IR (SMD = − 0.54, *p* < 0.001) (Supplementary Material S6, Figs. 22–25). No significant changes were found for insulin. These findings confirm the results of previous studies, highlighting the effect of AE on glucose metabolism. A meta-analysis by Mattioni Maturana et al. [18] found superior improvements in HbA1c in healthy populations and HOMA-IR in overweight and obese individuals for moderate-intensity continuous exercise compared to high-intensity interval exercise. Both types of exercise influence several glucose-regulation and fat-oxidation pathways, which together influence glucose homeostasis. Regardless of exercise intensity, our results in healthy and clinical populations show a tendency toward improvement in the aforementioned markers. Notably, while we did not find significant improvements in insulin levels, HOMA-IR significantly changed after AE. Though these factors cannot be used interchangeably, HOMA-IR is an indirect method of insulin resistance measurement. Consequently, the change in HOMA-IR might show improvements in insulin resistance even in the absence of significant changes when accounting for insulin specifically. Interestingly, the meta-regression results revealed a significant moderating effect of insulin on _ba_FMD improvement (*β* = 0.381, *p* = 0.034).

### Limitations and Future Directions

Several limitations of our study be acknowledged. First, we introduced several inclusion criteria in order to enhance the quality of the reported results. We focused only on studies reporting _ba_FMD, and the selected results reflect only studies incorporating this outcome and therefore excluded studies that assessed endothelial function via other means. We perceive the improved quality of the reported results as a strength, but we also acknowledge that this may be seen as a limitation, as we can only generalize our results to _ba_FMD. Second, it has been established that _ba_FMD tends to normalize in longer interventions (8–12 weeks) due to the structural remodeling process of the vascular wall [59]. Therefore, the pooled changes in _ba_FMD after intervention might not reflect the peak change in functional adaptations of the endothelium and vascular wall. Third, we included various populations, both healthy and those affected by noncommunicable diseases. Therefore, our results only show general tendencies in diverse populations, but might differ significantly for specific populations (i.e., diabetes, overweight, hypertension, CVD). We encourage referral to studies and meta-analyses that specifically investigate these populations for more detailed information. Finally, we used SMD in this meta-analysis for all variables, except _ba_FMD, due to significant methodological differences in measurement and devices used for their assessment. While this enabled pooled analysis of various studies, the use of SMD precluded us from reporting absolute changes in these factors. Nevertheless, we opted to also present pre- to post-intervention changes in secondary outcomes using MD in addition to SMD, to improve clinical interpretation of these outcomes. The pooled effects are presented in Supplementary material S6, Tables 5 and 6.

## Conclusion

Taken together, our findings underscore the interconnected nature of exercise-induced adaptations for the markers investigated in the current study. Improvements in endothelial function do not occur in isolation, but are rather the result of numerous molecular pathways influencing various aspects of health. Our results suggest concurrent improvements in _ba_FMD and several other clinically valuable outcomes after AE, including: hemodynamics, endothelial blood-derived biomarkers, CRF, body composition, the blood lipid profile, glucose metabolism, and the inflammatory response. Improvements in _ba_FMD are primarily modulated by improvements in body composition (i.e., body weight and body fat), followed by improvements in CRF, blood pressure regulation (i.e., SBP and DBP), and insulin levels. Further research is warranted to compare the effectiveness of different exercise types, intensities, and modalities and to better understand how they influence these variables.

## Supplementary Information

Supplementary material 1

## Data Availability

All datasets used during the current study are available from the corresponding authors upon reasonable request.

## Abbreviations

AE: Aerobic exercise
Aix: Augmentation index
_ba_FMD: Brachial-artery flow-mediated dilation
_ba_PWV: Brachial–ankle pulse wave velocity
BF%: Percent body fat
BFM: Body fat mass
BMI: Body mass index
BW: Body weight
_cf_PWV: Carotid–femoral pulse wave velocity
CRF: Cardiorespiratory fitness
CRP: C-reactive protein
CVD: Cardiovascular disease
DBP: Diastolic blood pressure
ET-1: Endothelin-1
FMD: Flow-mediated dilation
GET: Gas exchange threshold
HbA1c: Glycated hemoglobin
HDL: High-density lipoprotein
HOMA-IR: Homeostatic model assessment for insulin resistance
IL-6: Interleukin 6
LDL: Low-density lipoprotein
MAP: Mean arterial pressure
NO: Nitric oxide
NO_x_: Nitrogen oxides
PEI: Physical exercise intervention
RCP: Respiratory compensation point
RER: Respiratory exchange ratio
SBP: Systolic blood pressure
SMM: Skeletal muscle mass
tCHOL: Total cholesterol
TG: Triglycerides
TNF-α: Tumor necrosis factor-α
VEGF: Vascular endothelial growth factor
VO_2max_: Maximal oxygen uptake
