# Supplementary material for: Impact of Aerobic Exercise on Endothelial Function and Other Cardiovascular Disease Markers in Health and Disease: A Systematic Review and Meta-analysis with Meta-regression of Randomized Controlled Trials
Source: Sports Med Open. 2026 Oct 8;12:150. doi: 10.1186/s40798-026-01110-0 (PMC13650237; doi:10.1186/s40798-026-01110-0)
Supplement: Supplementary file 1 [file 40798_2026_1110_MOESM1_ESM.docx]

**Title: Impact of Aerobic Exercise on Endothelial Function and Other Cardiovascular Disease Markers in Health and Disease: A Systematic Review and Meta-analysis with Meta-regression of Randomized Controlled Trials**

**Running title: Aerobic exercise and endothelial function**

Authors: Simon Iskra^1^, Gasper Turnsek^1^, Kristina Drole^1^, Armin H. Paravlic^1,2,3^

^1^Faculty of Sport, University of Ljubljana, Ljubljana, Slovenia

^2^Science and Research Centre Koper, Institute for Kinesiology Research, Koper, Slovenia

^3^Faculty of Sports Studies, Incubator of Kinanthropology Research, Masaryk University, Brno, Czechia

**Corresponding author:*

Simon Iskra

Faculty of Sport, University of Ljubljana

Gortanova 22, 1000 Ljubljana, Slovenia

E-mail: Simon Iskra@fsp.uni-lj.si

**E-mail; ORCID:**

Simon Iskra: Simon Iskra@fsp.uni-lj.si; 0009-0008-9768-9153

***Supplementary File***

**Impact of Aerobic Exercise on Endothelial Function and Other Cardiovascular Disease Markers in Health and Disease: A Systematic Review and Meta-analysis with Meta-regression of Randomized Controlled Trials**

**Contents**

[S1 – Search strategy	5](#_Toc235005720)

[Cumulative Index to Nursing and Allied Health Literature (CINAHL)	5](#_Toc235005721)

[Cochrane Central Register of Controlled Trials (CENTRAL)	5](#_Toc235005722)

[EBSCOhost	5](#_Toc235005723)

[EMBASE	5](#_Toc235005724)

[PubMed	6](#_Toc235005725)

[Web of Science	6](#_Toc235005726)

[S2 –Methodology used for categorization of physical exercise interventions	6](#_Toc235005727)

[Supplementary Table 1. Indices of exercise intensity for endurance exercise from maximal exercise testing and training zones.	6](#_Toc235005728)

[S3 - Methodological quality assessment of individual studies	8](#_Toc235005729)

[Supplementary Table 2. Fulfilment of Physiotherapy Evidence Database (PEDro) criteria for each of the studies included in the present meta-analysis	8](#_Toc235005730)

[S4 - Exercise intervention characteristics	11](#_Toc235005731)

[Supplementary Table 3. Exercise intervention characteristics for individual studies.	11](#_Toc235005732)

[S5 - Risk of bias assessment	19](#_Toc235005733)

[Supplementary Table 4. Risk of bias assessment for individual studies.	19](#_Toc235005734)

[S6 - Results of the meta-analysis for secondary parameters in the included studies	23](#_Toc235005735)

[Supplementary Table 5. Summary and pooled effects of the meta-analysis secondary parameters using standardized mean difference (SMD) and random-effects model.	23](#_Toc235005736)

[Supplementary Table 6. Summary and pooled effects of the meta-analysis secondary parameters using mean difference (MD) and random-effects model.	25](#_Toc235005737)

[**Fig. 1** Changes in carotid-femoral pulse wave velocity after aerobic exercise	29](#_Toc235005738)

[**Fig. 2** Changes in brachial-ankle pulse wave velocity after aerobic exercise	30](#_Toc235005739)

[**Fig. 3** Changes in augmentation index after aerobic exercise	31](#_Toc235005740)

[**Fig. 4** Changes in mean arterial pressure after aerobic exercise	32](#_Toc235005741)

[**Fig. 5** Changes in systolic blood pressure after aerobic exercise	34](#_Toc235005742)

[**Fig. 6** Changes in diastolic blood pressure after aerobic exercise	37](#_Toc235005743)

[**Fig. 7** Changes in nitric oxide after aerobic exercise	38](#_Toc235005744)

[**Fig. 8** Changes in nitrogen oxides after aerobic exercise	39](#_Toc235005745)

[**Fig. 9** Changes in endothelin-1 after aerobic exercise	40](#_Toc235005746)

[**Fig. 10** Changes in VO_2max_ after aerobic exercise	42](#_Toc235005747)

[**Fig. 11** Changes in respiratory exchange ratio after aerobic exercise	43](#_Toc235005748)

[**Fig. 12** Changes in gas-exchange threshold after aerobic exercise	44](#_Toc235005749)

[**Fig. 13** Changes in body weight after aerobic exercise	46](#_Toc235005750)

[**Fig. 14** Changes in body fat mass after aerobic exercise	47](#_Toc235005751)

[**Fig. 15** Changes in body fat percentage after aerobic exercise	48](#_Toc235005752)

[**Fig. 16** Changes in body mass index after aerobic exercise	50](#_Toc235005753)

[**Fig. 17** Changes in skeletal muscle mass after aerobic exercise	51](#_Toc235005754)

[**Fig. 18** Changes in high-density lipoprotein after aerobic exercise	53](#_Toc235005755)

[**Fig. 19** Changes in low-density lipoprotein after aerobic exercise	53](#_Toc235005756)

[**Fig. 20** Changes in triglyceride after aerobic exercise	54](#_Toc235005757)

[**Fig. 21** Changes in total cholesterol after aerobic exercise	55](#_Toc235005758)

[**Fig. 22** Changes in glucose after aerobic exercise	57](#_Toc235005759)

[**Fig. 23** Changes in insulin after aerobic exercise	57](#_Toc235005760)

[**Fig. 24** Changes in glycated haemoglobin after aerobic exercise	58](#_Toc235005761)

[**Fig. 25** Changes in homeostatic model assessment for insulin resistance after aerobic exercise	59](#_Toc235005762)

[**Fig. 26** Changes in C-reactive protein after aerobic exercise	60](#_Toc235005763)

[**Fig. 27** Changes in tumor necrotic factor-α after aerobic exercise	61](#_Toc235005764)

[**Fig. 28** Changes in interleukin 6 after aerobic exercise	62](#_Toc235005765)

[References	63](#_Toc235005766)

# S1 – Search strategy

## Cumulative Index to Nursing and Allied Health Literature (CINAHL)

“endothelial function” AND (exercise or physical activity or fitness or aerobic training or strength training) AND (randomized controlled trials or rct or randomised control trial)

## Cochrane Central Register of Controlled Trials (CENTRAL)

("endothelial function index" OR "flow-mediated dilation" OR "vascular endothelium") AND ("exercise" OR "Physical activity") AND randomized controlled trial in All Text - (Word variations have been searched)

## EBSCOhost

AB ( (adult) AND (vascular endothelium OR endothelial function OR endothelial dysfunction OR endothelium-dependent vasodilatation OR vascular reactivity OR flow-mediated dilatation OR FMD) AND (exercise OR physical exercise OR training) ) AND TX ( randomised controlled trial or randomized controlled trial or rct )

## EMBASE

adult/exp AND (exercise/exp OR 'physical activity'/exp) AND (('vascular endothelium'/exp OR 'blood vessel endothelium' OR 'endangium' OR 'endothelium, blood vessel' OR 'endothelium, vascular' OR 'endovasculum' OR 'vascular endothelium') OR 'endothelial function'/exp OR ('flow-mediated dilation test'/exp OR 'FMD technique' OR 'FMD test' OR 'flow-mediated dilatation assessment' OR 'flow-mediated dilatation technique' OR 'flow-mediated dilatation test' OR 'flow-mediated dilation assessment' OR 'flow-mediated dilation technique' OR 'flow-mediated dilation test') OR 'endothelium dependent vasodilation'/exp OR 'endothelium dependent relaxation'/exp OR 'endothelium dependent vasodilatation'/exp) AND ('randomized controlled trial'/exp OR 'controlled trial, randomized' OR 'randomised controlled study' OR 'randomised controlled trial' OR 'randomized controlled study' OR 'randomized controlled trial' OR 'trial, randomized controlled')

## PubMed

Search: ((adult[MeSH Terms]) OR (adult[Title/Abstract])) AND ((vascular endothelium[MeSH Terms]) OR (vascular endothelium[Title/Abstract]) OR (endothelial function[Title/Abstract]) OR (endothelial dysfunction[Title/Abstract]) OR (flow-mediated dilatation[Title/Abstract]) OR (endothelium-dependent vasodilatation[Title/Abstract]) OR (vascular reactivity[Title/Abstract])) AND ((exercise[MeSH Terms]) OR (exercise[Title/Abstract]) OR (physical exercise[MeSH Terms]) OR (physical exercise[Title/Abstract]) OR (exercise training[MeSH Terms]) OR (exercise training[Title/Abstract])) AND (randomized controlled trial[Publication Type])

("adult"[MeSH Terms] OR "adult"[Title/Abstract]) AND ("endothelium, vascular"[MeSH Terms] OR "vascular endothelium"[Title/Abstract] OR "endothelial function"[Title/Abstract] OR "endothelial dysfunction"[Title/Abstract] OR "flow mediated dilatation"[Title/Abstract] OR "endothelium dependent vasodilatation"[Title/Abstract] OR "vascular reactivity"[Title/Abstract]) AND ("exercise"[MeSH Terms] OR "exercise"[Title/Abstract] OR "exercise"[MeSH Terms] OR "physical exercise"[Title/Abstract] OR "exercise"[MeSH Terms] OR "exercise training"[Title/Abstract]) AND "randomized controlled trial"[Publication Type]

## Web of Science

**ALL=((adult) AND (vascular endothelium OR endothelial function OR endothelial dysfunction OR endothelium-dependent vasodilatation OR vascular reactivity OR flow-mediated dilatation OR FMD) AND (exercise OR physical exercise OR training) AND (randomized controlled trial))**

# S2 –Methodology used for categorization of physical exercise interventions

## Supplementary Table 1. Indices of exercise intensity for endurance exercise from maximal exercise testing and training zones.

| Exercise intensity | VO_2max_ (%) | HR_max_ (%) | HRR (%) | RPE scale | Training Zone |
| --- | --- | --- | --- | --- | --- |
| Low | <40 | <55 | <40 | 10-11 | Aerobic |
| Moderate | 40-69 | 55-74 | 40-69 | 12-13 | Aerobic |
| High | 70-85 | 75-90 | 70-85 | 14-16 | Aerobic + lactate |
| Very high | >85 | >90 | >85 | 17-19 | Aerobic + lactate + anaerobic |

# S3 - Methodological quality assessment of individual studies

## Supplementary Table 2. Fulfilment of Physiotherapy Evidence Database (PEDro) criteria for each of the studies included in the present meta-analysis

| Study name | Criterion 1 | Criterion 2 | Criterion 3 | Criterion 4 | Criterion 5 | Criterion 6 | Criterion 7 | Criterion 8 | Criterion 9 | Criterion 10 | Criterion 11 | Total Score |
| --- | --- | --- | --- | --- | --- | --- | --- | --- | --- | --- | --- | --- |
| Hovespian et al. 2021 | YES | YES | YES | YES | NO | NO | YES | YES | NO | YES | YES | 7 |
| Stensvold et al. 2010 | YES | YES | YES | YES | NO | NO | NO | YES | NO | YES | YES | 6 |
| Shenouda et al. 2017 | YES | NO | YES | YES | NO | NO | NO | YES | NO | YES | YES | 5 |
| Kwon et al. 2011 | YES | YES | NO | YES | NO | NO | NO | NO | NO | YES | YES | 4 |
| Pugh et al. 2014 | YES | YES | YES | YES | YES | NO | NO | NO | NO | YES | YES | 6 |
| Robinson et al. 2016 | YES | NO | NO | YES | NO | NO | NO | NO | NO | YES | YES | 3 |
| Almenning et al. 2015 | YES | YES | NO | YES | NO | NO | NO | NO | NO | YES | YES | 4 |
| Beck et al. 2013 | YES | YES | NO | YES | NO | NO | NO | YES | NO | YES | YES | 5 |
| Boeno et al. 2020 | YES | YES | NO | YES | NO | NO | YES | YES | NO | YES | YES | 6 |
| McDermott et al. 2009 | YES | YES | NO | YES | NO | NO | YES | YES | YES | YES | YES | 7 |
| Vona et al 2009 | YES | YES | NO | YES | NO | NO | YES | YES | NO | YES | YES | 6 |
| Belardinelli et al. 2006 | NO | YES | NO | YES | NO | NO | YES | NO | NO | YES | YES | 5 |
| Belardinelli et al. 2005 | NO | YES | NO | YES | NO | NO | NO | YES | NO | YES | YES | 5 |
| Belardinelli et al. 2008 | NO | YES | NO | YES | NO | NO | YES | YES | NO | YES | YES | 6 |
| Benda et al. 2015 | YES | NO | NO | YES | NO | NO | NO | NO | NO | YES | YES | 3 |
| Eleuteri et al. 2013 | NO | YES | NO | YES | NO | NO | NO | YES | NO | YES | YES | 5 |
| Guazzi et al. 2004 | YES | YES | YES | YES | YES | NO | YES | YES | NO | YES | YES | 8 |
| Kitzman et al. 2013 | YES | YES | NO | YES | NO | NO | YES | YES | NO | YES | YES | 6 |
| Kobayashi et al. 2003 | NO | YES | NO | YES | NO | NO | YES | YES | NO | YES | YES | 6 |
| Sales et al. 2020 | YES | YES | NO | YES | NO | NO | NO | YES | NO | YES | YES | 5 |
| Turri-Silva et al. 2021 | YES | YES | YES | YES | NO | NO | YES | YES | NO | YES | YES | 7 |
| Van Craenenbroeck et al.2010 | YES | NO | NO | YES | NO | NO | NO | YES | YES | YES | YES | 5 |
| Wisløff et al. 2007 | YES | YES | NO | YES | NO | NO | NO | YES | NO | YES | YES | 5 |
| Lavrenčič et al. 2000 | YES | YES | NO | YES | NO | NO | NO | YES | NO | NO | YES | 4 |
| Vona et al. 2004 | YES | YES | NO | YES | NO | NO | YES | YES | NO | YES | YES | 6 |
| Blumenthal et al. 2005 | YES | YES | YES | YES | NO | NO | YES | YES | YES | YES | YES | 8 |
| Westhoff et al. 2007 | YES | YES | NO | YES | NO | NO | YES | YES | NO | YES | YES | 6 |
| Sixt et al. 2008 | NO | YES | NO | YES | NO | NO | YES | YES | NO | YES | NO | 5 |
| Westhoff et al. 2008 | YES | YES | NO | YES | NO | NO | NO | YES | NO | YES | YES | 5 |
| Desch et al. 2010 | NO | YES | NO | YES | NO | NO | NO | YES | NO | YES | YES | 5 |
| Hermann et al. 2011 | YES | YES | YES | YES | NO | NO | YES | YES | NO | YES | YES | 7 |
| Molmen-Hansen et al. 2012 | YES | YES | NO | YES | NO | NO | NO | NO | NO | YES | YES | 4 |
| Prakhinkit et al. 2014 | YES | YES | NO | YES | NO | NO | NO | YES | NO | YES | YES | 5 |
| Van Craenenbroeck et al. 2015 | YES | YES | YES | YES | NO | NO | YES | NO | NO | YES | YES | 6 |
| Sherwood et al. 2016 | YES | YES | NO | YES | NO | NO | NO | NO | YES | YES | YES | 5 |
| Azadpour et al. 2017 | YES | YES | NO | YES | NO | NO | NO | YES | NO | YES | YES | 5 |
| Ghardashi Afousi et al. 2018 | YES | YES | NO | YES | NO | NO | YES | NO | NO | YES | YES | 5 |
| Berroug et al. 2019 | YES | YES | NO | YES | NO | NO | YES | YES | NO | YES | YES | 6 |
| Boff et al. 2019 | YES | YES | YES | YES | NO | NO | YES | NO | NO | YES | YES | 6 |
| Bouaziz et al. 2019 | YES | YES | YES | YES | NO | NO | YES | YES | NO | YES | YES | 7 |
| Yoon et al. 2019 | YES | YES | NO | YES | NO | NO | NO | YES | NO | YES | YES | 5 |
| Isaksen et al. 2019 | YES | NO | NO | YES | NO | NO | YES | YES | NO | YES | YES | 5 |
| Kirkman et al. 2019 | YES | YES | NO | YES | NO | NO | NO | YES | NO | YES | YES | 5 |
| Lee et al. 2019 | YES | YES | NO | YES | NO | NO | NO | NO | NO | YES | YES | 4 |
| Claes 2020 | YES | YES | NO | YES | NO | NO | YES | NO | YES | YES | YES | 6 |
| Merlo et al. 2020 | YES | YES | NO | YES | NO | NO | NO | YES | NO | YES | YES | 5 |
| Blumenthal et al. 2021 | YES | YES | YES | YES | NO | NO | NO | YES | YES | YES | YES | 7 |
| Haynes et al. 2021 | YES | YES | NO | YES | NO | NO | YES | YES | NO | YES | YES | 6 |
| Davoodi et al. 2022 | YES | YES | NO | YES | NO | NO | YES | NO | NO | YES | YES | 5 |
| Collins et al. 2023 | YES | YES | YES | YES | NO | NO | YES | YES | NO | YES | YES | 7 |
| Hansen et al. 2023 | YES | YES | NO | YES | NO | NO | YES | YES | NO | YES | YES | 6 |
| Twerenbold et al. 2023 | YES | YES | NO | YES | NO | NO | YES | YES | NO | YES | YES | 6 |
| Braith et al. 2008 | NO | YES | NO | YES | NO | NO | YES | NO | NO | YES | YES | 5 |
| Jones et al. 2014 | NO | YES | YES | YES | NO | NO | YES | YES | NO | YES | YES | 7 |
| Belardinelli, Lacalaprice et al. 2008 | NO | YES | NO | YES | NO | NO | YES | YES | NO | YES | YES | 6 |
| Tjønna et al. 2008 | NO | YES | NO | YES | NO | NO | NO | NO | NO | YES | YES | 4 |
| Yoshizawa et al. 2010 | NO | YES | NO | YES | NO | NO | NO | NO | NO | YES | YES | 4 |
| Pierce et al. 2011 | YES | YES | NO | YES | NO | NO | YES | NO | NO | YES | YES | 5 |
| Abdi et al. 2021 | YES | YES | NO | YES | NO | NO | NO | YES | YES | YES | YES | 6 |
| He et al. 2022 | YES | YES | NO | YES | NO | NO | YES | YES | YES | YES | YES | 7 |
| Hwang et al. 2019 | YES | YES | NO | YES | NO | NO | NO | YES | YES | YES | YES | 6 |
| Suboc et al. 2014 | YES | YES | NO | YES | NO | NO | YES | YES | YES | YES | YES | 7 |
| Pereira et al. 2015 | YES | YES | NO | YES | NO | NO | NO | YES | YES | YES | YES | 6 |
| Eaungpulsawat e tal. 2023 | YES | YES | NO | YES | NO | NO | NO | YES | YES | YES | YES | 6 |
| Siripanya et al. 2023 | YES | YES | YES | YES | YES | NO | YES | YES | NO | YES | YES | 8 |
| Ploydang et al. 2023 | YES | YES | NO | YES | NO | NO | NO | YES | NO | YES | YES | 5 |
| Isaksen et al. 2014 | YES | NO | NO | YES | NO | NO | YES | YES | NO | YES | YES | 5 |
| Oliveira e Silva 2019 | NO | YES | NO | YES | NO | NO | YES | YES | YES | YES | YES | 7 |
| Aispuru-Lanche et al. 2024 [1] | YES | YES | YES | YES | NO | NO | NO | YES | YES | YES | YES | 7 |
| Goeder et al. 2024 | YES | YES | YES | YES | NO | NO | YES | YES | NO | YES | YES | 7 |
| Huang et al. 2025 | YES | YES | NO | YES | NO | NO | NO | YES | YES | YES | YES | 6 |
| Yu et al. 2025 | YES | YES | YES | YES | NO | NO | NO | YES | YES | YES | YES | 7 |

# S4 - Exercise intervention characteristics

Exercise intervention characteristics are presented in Supplementary Table 3.

## Supplementary Table 3. Exercise intervention characteristics for individual studies.

| Study | Intensity classification | Training type | Modality | Duration in weeks | Weekly frequency (times) | Weekly duration (min) | Reported Intensity |
| --- | --- | --- | --- | --- | --- | --- | --- |
| Hovespian et al. 2021 | high/very high | interval | ergometer-based | 10 | 4 | 160 | 85-90% HR_max_ |
| Stensvold et al. 2010 | high/very high | interval | treadmill | 12 | 3 | 129 | 90-95% HR_max_ |
| Shenouda et al. 2017 | high/very high | interval | cycling | 12 | 2.7 | 27 | “all-out” |
| Shenouda et al. 2017 | low/moderate | continuous | cycling | 12 | 2.7 | 121.5 | 70% HR_max_ |
| Kwon et al. 2011 | low/moderate | continuous | walking | 12 | 5 | 300 | 4-6 MET |
| Pugh et al. 2014 | low/moderate | continuous | ergometer-based | 16 | 3.5 | 135.1 | 30-60% HRR |
| Robinson et al. 2016 | high/very high | continuous | treadmill | 8 | 3 | 112.5 | 75% HR_max_ |
| Almenning et al. 2015 | high/very high | interval | walk/run/cycle | 10 | 3 | 120 | 90-95% HR_max_ |
| Beck et al. 2013 | high/very high | continuous | treadmill | 8 | 3 | 180 | 65-85% HR_max_ |
| Boeno et al. 2020 | high/very high | continuous | treadmill | 12 | 3 | 180 | 60-80% HRR |
| McDermott et al. 2009 | low/moderate | continuous | treadmill | 24 | 3 | 82.5 | 12-14 Borg scale |
| Vona et al 2009 | low/moderate | continuous | cycling | 4 | 4 | 240 | 75% HR_max_ |
| Belardinelli et al. 2006 a | low/moderate | continuous | cycling | 8 | 3 | 120 | 60% VO_2peak_ |
| Belardinelli et al. 2006 b | low/moderate | continuous | Cycling | 8 | 3 | 120 | 60% VO_2peak_ |
| Belardinelli et al. 2005 | low/moderate | continuous | Cycling | 8 | 3 | 120 | 60% VO_2peak_ |
| Belardinelli et al. 2008 a | high/very high | continuous | cycling and treadmill | 8 | 3 | 90 | 70% VO_2peak_ |
| Belardinelli et al. 2008 b | high/very high | interval | dancing | 8 | 3 | 63 | 70% VO_2peak_ |
| Benda et al. 2016 a | Low/high or low/very high | interval | cycling | 12 | 2 | 100 | 90% W_max_, 15-17 Borg scale |
| Benda et al. 2016 b | high/very high | continuous | cycling | 12 | 2 | 90 | 60-75% W_max_, 12-14 Borg scale |
| Eleuteri et al. 2013 | low/moderate | continuous | cycling | 12 | 5 | 150 | Ventilatory aerobic treshold |
| Guazzi et al. 2004 | moderate-high | continuous | cycling | 8 | 4 | 160 | 60-80% HRR |
| Kitzman et al. 2013 | low/moderate | continuous | walking, cycling, upper extremity ergometry (aerodyne arms only) | 16 | 3 | 180 | 40-70% HRR |
| Kobayashi et al. 2003 | low/moderate | continuous | cycling | 12 | 2.5 | 75 | HR at ventilatory aerobic threshold, 13 Borg |
| Sales et al. 2020 a | high/very high | interval | cycling | 12 | 3 | NR | 5% above the respiratory compensation point |
| Sales et al. 2020 b | moderate-high | continuous | cycling | 12 | 3 | NR | HR between the anaerobic threshold and respiratory compensation point |
| Turri-Silva et al. 2021 | moderate-high | interval | walking, cycling, upper extremity ergometry (aerodyne arms only) | 12 | 3 | 150 | HR between upper limit of moderate-intensity domain and 10% above based on CPET |
| Van Craenenbroeck et al. 2010 | high/very high | continuous | Not reported per se | 24 | 3 | 180 | 90% HR at respiratory compensation point |
| Wisløff et al. 2007 a | high/very high | interval | treadmill walking | 12 | 3 | 114 | 90-95% HR_max_ |
| Wisløff et al. 2007 b | low/moderate | continuous | treadmill walking | 12 | 3 | 141 | 70-75% HR_max_ |
| Lavrenčič et al 2000 | high/very high | continuous | cycling | 12 | 3 | 150 | 80% HR_max_ |
| Vona et al. 2004 | low/moderate | continuous | cycling | 12 | 3 | 180 | 75% HR_max_ |
| Blumenthal et al. 2005 | high/very high | continuous | walking, jogging | 16 | 3 | 105 | 70-85% HRR |
| Westhoff et al. 2007 | low/moderate | interval | walking | 12 | 3 | 76.5 | 1.8-2.2 mmol/L lactate |
| Sixt et al. 2008 | low/moderate | continuous | cycling | 4 | 7 | 330 | 70% HR_max_ |
| Westhoff et al. 2008 | low/moderate | interval | arm-cycling | 12 | 3 | 60 | 2 mmol/L lactate |
| Desch et al. 2010 | high/very high | continuous | cycling and group exercise sessions | 25 | 7 | 390 | 75% HR_max_ |
| Herman et al. 2011 | high/very high | interval | cycling, staircase running | 8 | 3 | 156 | 80-90% VO_2peak_ |
| Molmen-Hansen et al. 2012 a | low/moderate | continuous | treadmill walking/running | 12 | 3 | 141 | 70% HR_max_, 60% VO_2peak_ |
| Molmen-Hansen et al. 2012 b | high/very high | interval | treadmill walking/running | 12 | 3 | 114 | 90-95% HR_max_, 85-90% VO_2peak_ |
| Prakhinkit et al. 2014 a | low/moderate | continuous | walking | 12 | 3 | 75 | 20-50% HRR |
| Prakhinkit et al. 2014 b | low/moderate | continuous | walking | 12 | 3 | 75 | 20-50% HRR |
| Van Craenenbroeck et al. 2015 | high/very high | continuous | cycling | 12 | 28 | 280 | 90% HR at anaerobic threshold |
| Sherwood et al. 2016 a | high/very high | continuous | treadmill walking or running | 16 | 3 | 90 | 70-85% HRR |
| Sherwood et al. 2016 b | high/very high | continuous | treadmill walking or running | 16 | 3 | 90 | 70-85% HRR |
| Azadpour et al. 2017 | low/moderate | continuous | treadmill walking or jogging | 10 | 3 | 97.5 | 50-70% HRR |
| Ghardashi Afousi et al. 2018 a | high/very high | interval | Cycling | 12 | 3 | 180 | 85-90% HR_max_ |
| Ghardashi Afousi et al. 2018 b | low/moderate | continuous | Cycling | 12 | 3 | 186 | 70% HR_max_ |
| Berroug et al. 2019 | low/moderate | continuous | Walking | 26 | 3 | 150 | Up to maximal ischemic leg symptoms |
| Boff et al. 2019 a | moderate-high | interval | Cycling | 8 | 3 | 120 | 80-85% HR_max_ |
| Boff et al. 2019 b | low/moderate | continuous | Cycling | 8 | 3 | 120 | 60-65% HR_max_ |
| Bouaziz et al. 2019 | low/moderate | interval | Cycling | 9.5 | 2 | 60 | First ventilatory threshold workload |
| Yoon et al. 2019 | low/moderate | continuous | Walking | 12 | 3 | 90 | 40-60% HRR |
| Isaksen et al. 2019 | high/very high | interval | Cycling or running | 12 | 3 | 180 | 85% HR_max_ |
| Kirkman et al. 2019 | moderate-high | continuous | Cycling/jogging/elliptical trainer | 12 | 3 | 135 | 60-85% HRR, 12-16 Borg scale |
| Lee et al. 2019 | high/very high | interval | Cycling | 8 | 3 | 90 | W_max_ |
| Claes et al. 2020 | low/moderate | continuous | Multi-level cardiac rehabilitation program | NR | NR | 150 | PATHway multi-level cardiac rehabilitation program |
| Merlo et al. 2020 | low/moderate | continuous | Treadmill running or walking | 8 | 2 | 30 | 60-70% HR_max_, 5-6 out of 10 Borg scale |
| Blumenthal et al. 2021 | high/very high | continuous | Walking, jogging or cycling | 12 | 3 | 150 | 70-85% HRR |
| Haynes et al. 2021 a | low/moderate | continuous | Land walking | 24 | 3 | 97.5 | 40-65% HRR |
| Haynes et al. 2021 b | low/moderate | continuous | Water walking | 24 | 3 | 82.5 | 40-65% HRR |
| Davoodi et al. 2022 a | high/very high | interval | cycling | 12 | 3 | 186 | 85-90% HR_max_ |
| Davoodi et al. 2022 b | low/moderate | continuous | cycling | 12 | 3 | 186 | 70% HR_max_ |
| Collins et al. 2023 a | low/moderate | continuous | Cycling | 12 | 3 | 165 | 60-70% HR_max_ |
| Collins et al. 2023 b | high/very high | interval | Cycling | 12 | 3 | 78 | 85% HR_max_ |
| Collins et al. 2023 c | high/very high | interval | Cycling | 12 | 3 | 78 | 85% HR_max_ |
| Hansen et al. 2023 | moderate-high | continuous | Rowing ergometry | 12 | 3 | 90 | 12-17 Borg scale |
| Twerenbold et al. 2023 | high/very high | interval | Cycling | 8 | 3 | 135 | 80-95% HR_max_ |
| Braith et al. 2008 | low/moderate | continuous | treadmill walking | 12 | 3 | 105 | 11-14 Borg scale |
| Jones et al. 2014 | Low/high or low/very high | interval | treadmill walking | 25 | 5 | 37.5 | 55-100% VO_2peak_ |
| Belardinelli et al. 2008 | low/moderate | continuous | Not specified | 8 | 3 | NR | 60% VO_2peak_ |
| Tjønna et al. 2008 a | high/very high | continuous | Treadmill walking or running | 16 | 3 | 141 | 70% HR_max_ |
| Tjønna et al. 2008 b | high/very high | interval | Treadmill walking or running | 16 | 3 | 120 | 90% HR_max_ |
| Yoshizawa et al. 2010 | low/moderate | continuous | Walking or cycling | 8 | 4 | 150 | 60-75% HR_max_ |
| Pierce et al. 2011 | moderate-high | continuous | Walking | 8 | 4 | 295 | 75% HR_max_ |
| Abdi et al. 2021 | high/very high | interval | treadmill running | 12 | 3 | 120 | 85-95% HR_max_ |
| He et al. 2022 a | low/moderate | continuous | treadmill running | 4 | 5 | 250 | 40% HRR |
| He et al. 2022 b | moderate-high | continuous | treadmill running | 4 | 3 | 120 | 70-80% HR_max_ |
| He et al. 2022 c | Low/high or low/very high | interval | treadmill running | 4 | 3 | 90 | 85-90% HR_max_ |
| He et al. 2022 d | low/moderate | continuous | treadmill running | 8 | 5 | 250 | 40% HRR |
| He et al. 2022 e | moderate-high | continuous | treadmill running | 8 | 3 | 120 | 70-80% HR_max_ |
| He et al. 2022 f | Low/high or low/very high | interval | treadmill running | 8 | 3 | 90 | 85-90% HR_max_ |
| He et al. 2022 g | low/moderate | continuous | treadmill running | 12 | 5 | 250 | 40% HRR |
| Hwang et al. 2019 a | high/very high | interval | Arm and leg ergometry | 8 | 4 | 160 | 90% HR_max_ |
| Hwang et al. 2019 b | low/moderate | continuous | Arm and leg ergometry | 8 | 4 | 188 | 70% HR_max_ |
| Suboc et al. 2014 a | low/moderate | continuous | Walking | 12 | NR | NR | Pedometer guidance |
| Suboc et al. 2014 b | low/moderate | continuous | Walking | 12 | NR | NR | Pedometer guidance |
| Pereira et al. 2015 | low/moderate | continuous | Walking | 4 | 5 | 225 | Brisk walking |
| Ploydang et al. 2023 | low/moderate | continuous | Nordic walking in water | 12 | 3 | 120 | 40-60% HRR |
| Isaksen et al. 2014 | moderate-high | interval | Cycling ergometer or treadmill running | 12 | 3 | 180 | 85% HR_max_, 15-17 Borg scale |
| Eaungpulsawat et al. 2023 | low/moderate | continuous | Walking, rising from a chair | 12 | 3 | NR | 50-70% HR_max_ |
| Siripanya et al. 2023 | low/moderate | continuous | Buddhist walking | 12 | 3 | 112.5 | 41-60% HRR |
| Oliveira e Silva et al. 2019 | low/moderate | continuous | cycling | 16 | 3 | 90 | 65-75% HR_max_, 13 Borg scale |
| Aispuru-Lanche et al. 2024 a | high/very high | interval | Cycling, running | 16 | 2 | 40 | Above second ventilatory threshold |
| Aispuru-Lanche et al. 2024 b | high/very high | interval | Cycling, running | 16 | 2 | 60 | Above second ventilatory threshold |
| Goeder et al. 2024 | moderate-high | continuous | cycling | 8 | 3 | 90 | 40-50% VO_2peak_, progressively higher |
| Huang et al. 2025 a | high/very high | interval | cycling | 8 | 3 | 75 | 90% VO_2peak_ |
| Huang et al. 2025 b | low/moderate | continuous | cycling | 8 | 3 | 120 | 60% VO_2peak_ |
| Yu et al. 2025 a | high/very high | interval | swimming | 8 | 3 | 132 | 85–95% HR_max_ |
| Yu et al. 2025 b | low/moderate | continuous | swimming | 8 | 3 | 165 | 70–75% HR_max_ |
| Note: CPET – cardio-pulmonary exercise test; HR – heart rate; HR_max_ – maximal heart rate; HRR – heart rate reserve; MET – metabolic equivalent; VO_2peak_ – peak oxygen uptake; W_max_ – maximal achieved workload during cardio-pulmonary exercise test. | | | | | | | |

# S5 - Risk of bias assessment

Individual studies risk of bias assessment results are presented in Supplementary Table 4.

## Supplementary Table 4. Risk of bias assessment for individual studies.

| **Study name** | **D1** | **D2** | **D3** | **D4** | **D5** | **Overall bias** |
| --- | --- | --- | --- | --- | --- | --- |
| Hovespian et al. 2021 | High | Some concerns | Low | Low | Low | High |
| Stensvold et al. 2010 | Low | Low | Low | Low | Low | Low |
| Shenouda et al. 2017 | Some concerns | Low | Low | Low | Low | Some concerns |
| Kwon et al. 2011 | Low | Low | Low | Low | Low | Low |
| Pugh et al. 2014 | Low | Low | Low | Low | Low | Low |
| Robinson et al. 2016 | High | Low | Low | Some concerns | Low | High |
| Almenning et al. 2015 | Low | Some concerns | Low | Low | Low | Some concerns |
| Beck et al. 2013 | Low | Low | Low | Low | Low | Low |
| Boeno et al. 2020 | Low | Low | Low | Low | Low | Low |
| McDermott et al. 2009 | Low | Low | Low | Low | Low | Low |
| Vona et al. 2009 | Low | High | High | Low | Low | High |
| Belardinelli et al. 2006 | Low | Low | Low | Low | Low | Low |
| Belardinelli et al. 2005 | Some concerns | Low | Low | Low | Low | Some concerns |
| Belardinelli et al. 2008 | Low | Some concerns | Low | Low | Low | Some concerns |
| Benda et al. 2015 | High | Some concerns | Low | Low | Low | High |
| Eleuteri et al. 2013 | Low | Some concerns | Low | Low | Low | Some concerns |
| Guazzi et al. 2004 | Some concerns | Some concerns | Low | Low | Low | Some concerns |
| Kitzman et al. 2013 | Low | Some concerns | Low | Low | Low | Some concerns |
| Kobayashi et al. 2007 | Low | Low | Low | Low | Low | Low |
| Sales et al. 2020 | Some concerns | Low | Low | Low | Low | Some concerns |
| Turri-Silva et al. 2021 | Low | Some concerns | Low | Low | Low | Some concerns |
| Van Craernenbroeck et al. 2010 | Some concerns | Some concerns | Low | Low | Low | Some concerns |
| Wisloff et al. 2007 | Low | Some concerns | Low | Low | Low | Some concerns |
| Lavrenčič et al. 2000 | Some concerns | Low | Low | Low | Low | Some concerns |
| Vona et al. 2004 | Low | Some concerns | Low | Low | Low | Some concerns |
| Blumenthal et al. 2005 | Low | Low | Low | Low | Low | Low |
| Westhoff et al. 2007 | Low | Low | Low | Low | Low | Low |
| Sixt et al. 2008 | Some concerns | Low | Low | Low | Low | Some concerns |
| Westhoff et al. 2008 | Low | Low | Low | Low | Low | Low |
| Desch et al. 2010 | Low | Low | Low | Low | Low | Low |
| Hermann et al. 2011 | Low | Low | Low | Low | Low | Low |
| Molmern-Hansen et al. 2012 | Low | Some concerns | Low | Low | Low | Some concerns |
| Prakhinkit et al. 2014 | Some concerns | Low | Low | Low | Low | Some concerns |
| Van Craenenbroeck et al. 2015 | Low | Low | Low | Low | Low | Low |
| Sherwood et al. 2016 a | Some concerns | Low | Low | Low | Low | Some concerns |
| Azadpour et al. 2017 | Some concerns | Low | Low | Low | Low | Some concerns |
| Ghardashi Afousi et al. 2018 | Low | Low | Low | Low | Low | Low |
| Berroug et al. 2019 | Some concerns | Some concerns | Low | Low | Low | Some concerns |
| Boff et al. 2019 | High | Some concerns | Low | Low | Low | High |
| Bouaziz et al. 2019 | Low | Low | Low | Low | Low | Low |
| Yoon et al. 2019 | High | Some concerns | Some concerns | Low | Some concerns | High |
| Isaksen et al. 2019 | High | Some concerns | Low | Low | Low | High |
| Kirkman et al. 2019 | Low | Low | Low | Low | Low | Low |
| Lee et al. 2019 | Low | Low | Low | Low | Low | Low |
| Claes et al. 2020 | Low | Low | Low | Low | Low | Low |
| Merlo et al. 2020 | Low | Low | Low | Low | Low | Low |
| Blumenthal et al. 2021 | Low | Low | Low | Low | Low | Low |
| Haynes et al. 2021 | Some concerns | Low | Low | Low | Low | Some concerns |
| Davoodi et al. 2022 | Some concerns | Some concerns | Low | Low | Low | Some concerns |
| Collins et al. 2023 | Low | Low | Low | Low | Low | Low |
| Hansen et al. 2023 | Some concerns | Some concerns | Low | Low | Low | Some concerns |
| Twerenbold et al. 2023 | Low | Low | Low | Low | Low | Low |
| Braith et al. 2008 | Some concerns | Low | Low | Low | Low | Some concerns |
| Jones et al. 2014 | Low | Low | Low | Low | Low | Low |
| Belardinelli, Lacalaprice et al. 2008 | Low | Low | Low | Low | Low | Low |
| Tjonna et al. 2008 | Low | Low | Low | Low | Low | Low |
| Yoshiyawa et al. 2010 | Low | Low | Low | Low | Low | Low |
| Pierce et al. 2011 | Some concerns | Low | Low | Low | Low | Some concerns |
| Abdi et al. 2021 | Some concerns | Low | Low | Low | Low | Some concerns |
| He et al. 2022 | Some concerns | Low | Low | Low | Low | Some concerns |
| Hwang et al. 2019 | Some concerns | High | Low | Some concerns | Some concerns | High |
| Suboc et al. 2014 | Low | Low | Low | Low | Low | Low |
| Pereira et al. 2015 | Some concerns | High | High | High | Some concerns | High |
| Eaungpulsawat et al. 2023 | Some concerns | Low | Low | Low | Low | Some concerns |
| Siripanya et al. 2023 | Low | Low | Low | Low | Low | Low |
| Oliveira e Silva et al. 2019 | Low | Low | Low | Low | Low | Low |
| Ploydang et al. 2023 | Low | Low | Low | Low | Low | Low |
| Isaksen et al. 2014 | High | Low | Low | Low | Low | High |
| Aispuru-Lanche et al. 2024 | Low | Low | Low | Low | Low | Low |
| Goeder et al. 2024 | Low | Low | Some concerns | Low | Low | Some concerns |
| Huang et al. 2024 | Low | Low | Low | Low | Low | Low |
| Yu et al. 2025 | Low | Low | Low | Low | Low | Low |
| D1 – Randomization process; D1 – Deviations from intended interventions; D3 – Missing outcome data; D4 – Measurement of the outcome; D5 Selection of the reported result. | | | | | | |

# S6 - Results of the meta-analysis for secondary parameters in the included studies

Supplementary Table 5 presents the magnitude and direction of effects with calculated pooled summary estimates of standardized mean difference (SMD) and 95% confidence intervals (CI), along with study heterogeneity statistics (I^2^).

## Supplementary Table 5. Summary and pooled effects of the meta-analysis secondary parameters using standardized mean difference (SMD) and random-effects model.

| Parameter | N | Effect | SMD | Lower 95% CI | Upper 95% CI | p-value | I^2^ |
| --- | --- | --- | --- | --- | --- | --- | --- |
| Haemodynamic measurements | | | | | | | |
| _cf_PWV | 13 | trivial | -0.03 | -0.23 | 0.17 | 0.778 | 20.23 |
| _ba_PWV | 5 | small | -0.53 | -1.01 | -0.05 | 0.03* | 32.99 |
| AIx | 9 | trivial | -0.10 | -0.33 | 0.12 | 0.3701 | 15.98 |
| MAP | 10 | small | -0.6 | -0.99 | -0.21 | 0.003* | 59.31 |
| SBP | 55 | small | -0.35 | -0.52 | -0.18 | 0.0001* | 70.15 |
| DBP | 53 | small | -0.41 | -0.61 | -0.21 | 0.0001* | 75.64 |
| Endothelial blood markers | | | | | | | |
| NO | 7 | large | 1.23 | 0.56 | 1.89 | 0.0003* | 76.71 |
| NO_x_ | 6 | large | 1.23 | 0.75 | 1.7 | 0.0001* | 51.09 |
| ET-1 | 7 | moderate | -0.99 | -1.49 | -0.48 | 0.0001* | 51.09 |
| Cardiorespiratory fitness | | | | | | | |
| VO_2max_ | 54 | moderate | 0.81 | 0.64 | 0.97 | 0.0001* | 65.10 |
| GET | 7 | moderate | 0.85 | -0.19 | 1.89 | 0.11 | 93.3 |
| RER | 10 | trivial | 0.01 | -0.16 | 0.18 | 0.923 | 0 |
| Body composition | | | | | | | |
| BW | 40 | trivial | -0.13 | -0.24 | -0.17 | 0.0241* | 0 |
| BFM | 11 | small | -0.24 | -0.44 | -0.03 | 0.0241 | 0 |
| BF% | 19 | small | -0.36 | -0.56 | -0.17 | 0.0003* | 27.69 |
| BMI | 49 | trivial | -0.19 | -0.28 | -0.10 | 0.0001* | 0 |
| SMM | 10 | trivial | 0.12 | -0.12 | 0.35 | 0.3387 | 0 |
| Blood lipid profile | | | | | | | |
| HDL | 43 | small | 0.31 | 0.11 | 0.52 | 0.003* | 74.63 |
| LDL | 39 | small | -0.23 | -0.33 | -0.12 | 0.0001* | 0 |
| TG | 40 | small | -0.19 | -0.32 | -0.07 | 0.0027* | 22.2 |
| t-CHO | 38 | trivial | 0.02 | -0.2 | 0.24 | 0.843 | 76.82 |
| Glucose metabolism | | | | | | | |
| Glucose | 28 | small | -0.27 | -0.43 | -0.11 | 0.0008 | 27.81 |
| Insulin | 16 | trivial | -0.19 | -0.67 | 0.28 | 0.4294 | 87.04 |
| HbA1c | 11 | small | -0.35 | -0.63 | -0.07 | 0.0147* | 28.97 |
| HOMA-IR | 16 | small | -0.54 | -0.83 | -0.25 | 0.0003* | 64.01 |
| Inflammation markers | | | | | | | |
| CRP | 20 | small | -0.36 | -0.61 | -0.11 | 0.0049* | 54.55 |
| TNF-alpha | 7 | small | -0.39 | -0.87 | 0.10 | 0.1186 | 60.98 |
| IL-6 | 7 | trivial | -0.17 | -0.48 | 0.15 | 0.2996 | 13.61 |
| * Indicates statistical significance. SMD – standardized mean difference; CI –confidence interval ; _cf_PWV – carotid-femoral pulse-wave velocity; _ba_PWV – brachial-ankle pulse-wave velocity; Aix – augmentation index; MAP – mean arterial pressure; SBP – systolic blood pressure; DBP – diastolic blood pressure; NO – nitric oxide; NO_x_ – nitrogen oxides; ET-1 – endothelin-1; VO_2max_ – maximal oxygen uptake; GET – gas-exchange threshold; RER – respiratory exchange ratio; BW – body weight; BFM – body fat mass; PBF – percent body fat; BMI – body mass index; SMM – skeletal muscle mass; HDL – high-density lipoprotein; LDL – low-density lipoprotein; TG – triglycerides; t-CHO – total cholesterol; HbA1c – glycated haemoglobin; HOMA-IR – homeostatic model assessment of insulin resistance; CRP – C-reactive protein; TNF-α – tumor necrosis factor-α; IL-6 – interleukin-6. | | | | | | | |

Supplementary Table 6 presents the magnitude and direction of effects with calculated pooled summary estimates of mean difference (MD) and 95% confidence intervals (CI), along with study heterogeneity statistics (I^2^).

| Supplementary Table 6. Summary and pooled effects of the meta-analysis secondary parameters using mean difference (MD) and random-effects model.Parameter | N | MD | Lower 95% CI | Upper 95% CI | p-value | I^2^ |
| --- | --- | --- | --- | --- | --- | --- |
| Haemodynamic measurements | | | | | | |
| _cf_PWV (m/s) | 13 | -0.01 | -0.26 | 0.24 | 0.9493 | 37.3 |
| _ba_PWV (m/s) | NA | NA | NA | NA | NA | NA |
| Aix (%) | 9 | -1.42 | -2.68 | -0.16 | 0.0276* | 0.00 |
| MAP (mm Hg) | 10 | -5.78 | -8.56 | -2.99 | < 0.0001* | 37.39 |
| SBP (mm Hg) | 55 | -3.94 | -5.52 | -2.36 | < 0.0001* | 81.49 |
| DBP (mm Hg) | 53 | -2.55 | -3.79 | -1.30 | < 0.0001* | 79.79 |
| Endothelial blood markers | | | | | | |
| NO (μmol/L) | 7 | 6.00 | 0.42 | 11.59 | 0.0352* | 99.29 |
| NO_x_ (μmol/L) | 6 | 4.05 | 2.04 | 6.06 | < 0.001* | 79.61 |
| ET-1 (pg/mL) | 7 | -0.55 | -0.84 | -0.27 | 0.0002* | 55.26 |
| Cardiorespiratory fitness | | | | | | |
| VO_2max_  (mlO_2_.kg^-1^.min^-1^) | 54 | 3.06 | 2.67 | 3.45 | < 0.0001* | 29.90 |
| GET (mlO_2_.kg^-1^.min^-1^) | 7 | 1.61 | 0.71 | 2.51 | 0.0004* | 58.19 |
| RER | 10 | 0.002 | -0.02 | 0.03 | 0.9180 | 31.91 |
| Body composition | | | | | | |
| BW (kg) | 40 | -1.36 | -2.53 | -0.19 | 0.0227* | 0.00 |
| BFM (kg) | 11 | -1.76 | -2.89 | -0.63 | 0.0023* | 0.00 |
| BF% (%) | 19 | -2.14 | -3.10 | -1.17 | < 0.0001* | 27.94 |
| BMI (kg/m^2^) | 49 | -0.72 | -1.01 | -0.42 | < 0.0001* | 16.22 |
| SMM (kg) | 10 | 0.96 | 0.05 | 1.88 | 0.0392* | 0.00 |
| Blood lipid profile | | | | | | |
| HDL (mmol/L) | 43 | 0.07 | 0.03 | 0.10 | < 0.0001* | 78.11 |
| LDL (mmol/L) | 39 | -0.17 | -0.25 | -0.08 | < 0.0001* | 15.78 |
| TG (mmol/L) | 40 | -0.08 | -0.15 | -0.02 | 0.0070* | 36.14 |
| t-CHO (mmol/L) | 38 | -0.05 | -0.15 | 0.06 | 0.4122 | 76.13 |
| Glucose metabolism | | | | | | |
| Glucose (mmol/L) | 28 | -0.23 | -0.39 | -0.07 | 0.0050 | 37.73 |
| Insulin (μIU/mL) | 16 | 0.03 | -2.97 | 3.03 | 0.9829 | 95.65 |
| HbA1c (%) | 11 | -0.35 | -0.64 | -0.04 | 0.0254 | 47.44 |
| HOMA-IR (%) | 16 | -0.73 | -1.09 | -0.37 | < 0.0001* | 61.08 |
| Inflammation markers | | | | | | |
| CRP (mg/L) | 20 | -2.12 | -3.99 | -0.25 | 0.0263 | 91.68 |
| TNF-alpha (pg/mL) | 7 | -0.72 | -1.56 | 0.11 | 0.0888 | 44.95 |
| IL-6 (pg/mL) | 7 | -0.19 | -0.40 | 0.03 | 0.0843 | 9.01 |
| * Indicates statistical significance. MD –mean difference; CI –confidence interval ; _cf_PWV – carotid-femoral pulse-wave velocity; _ba_PWV – brachial-ankle pulse-wave velocity; Aix – augmentation index; MAP – mean arterial pressure; SBP – systolic blood pressure; DBP – diastolic blood pressure; NO – nitric oxide; NO_x_ – nitrogen oxides; ET-1 – endothelin-1; VO_2max_ – maximal oxygen uptake; GET – gas-exchange threshold; RER – respiratory exchange ratio; BW – body weight; BFM – body fat mass; PBF – percent body fat; BMI – body mass index; SMM – skeletal muscle mass; HDL – high-density lipoprotein; LDL – low-density lipoprotein; TG – triglycerides; t-CHO – total cholesterol; HbA1c – glycated haemoglobin; HOMA-IR – homeostatic model assessment of insulin resistance; CRP – C-reactive protein; TNF-α – tumor necrosis factor-α; IL-6 – interleukin-6. | | | | | | |

***Effects of aerobic exercise on haemodynamic measurements***

In total, 12 independent comparisons were included in the _cf_PWV analysis. Meta-analysis demonstrated a *trivial,* non-significant decrease in _cf_PWV after AE (SMD = -0.03; 95% CI -0.23 to 0.17; p = 0.7777; I^2^ = 20.23%). A similar effect was observed when the fixed-effect model was used (SMD = -0.03; 95% CI -0.20 to 0.15; p = 0.7793; I^2^ = 30.28%). Following the exclusion of one study [2], the random-effects model produced a similar magnitude of effect (SMD = -0.06; 95% CI -0.26 to 0.13; p = 0.5321; I^2^ = 13.02%), supporting the robustness of the overall effect estimate. To facilitate clinical interpretation, the corresponding pooled effect, expressed as a mean difference was -0.01 m/s and is presented in the Supplementary Table 6.

## Fig. 1 Changes in carotid-femoral pulse wave velocity after aerobic exercise

In total, 5 independent comparisons were included in the _ba_PWV analysis. Meta-analysis demonstrated a *small* significant decrease in _ba_PWV after AE (SMD = -0.53; 95% CI -1.01 to -0.05; p = 0.0300; I^2^ = 32.99%). A similar effect was observed when the fixed-effect model was used (SMD = -0.53; 95% CI -0.92 to -0.14; p = 0.0075; I^2^ = 29.29%). Following the exclusion of one study [2], the random-effects model produced a *small*, non-significant magnitude of effect (SMD = -0.45; 95% CI -1.00 to 0.10; p = 0.1057; I^2^ = 40.61%). These discrepancies following exclusion of one study highlight the substantial heterogeneity and emphasize the need for cautious interpretation.

## Fig. 2 Changes in brachial-ankle pulse wave velocity after aerobic exercise

In total, 9 independent comparisons were included in the Aix analysis. Meta-analysis demonstrated a *trivial,* non-significant decrease in Aix after AE (SMD = -0.10; 95% CI -0.33 to 0.12; p = 0.3701; I^2^ = 15.98%). An identical effect was observed when the fixed-effect model was used (SMD = -0.09; 95% CI -0.29 to 0.12; p = 0.4098; I^2^ = 13.13%). Following the exclusion of one study [3], the random-effects model produced a similar magnitude of effect (SMD = -0.03; 95% CI -0.25 to 0.19; p = 0.7960; I^2^ = 0.0%), supporting the robustness of the overall effect estimate. To facilitate clinical interpretation, the corresponding pooled effect, expressed as a mean difference was -1.42% and is presented in the Supplementary Table 6.

## Fig. 3 Changes in augmentation index after aerobic exercise

In total, 10 independent comparisons were included in the MAP analysis. Meta-analysis demonstrated a *small* significant decrease in MAP after AE (SMD = -0.60; 95% CI -0.99 to -0.21; p = 0.0029; I^2^ = 59.31%). A *moderate* significant decrease was observed when the fixed-effect model was used (SMD = -0.65; 95% CI -0.89 to -0.40; p = 0.0001; I^2^ = 58.82%). Following the exclusion of one study [4], the random-effects model produced moderate significant effect (SMD = -0.48; 95% CI -0.81 to -0.15; p = 0.0049; I^2^ = 37.20%). These discrepancies between models highlight the substantial heterogeneity and emphasize the need for cautious interpretation. To facilitate clinical interpretation, the corresponding pooled effect, expressed as a mean difference was -5.78 mmHg and is presented in the Supplementary Table 6.

## Fig. 4 Changes in mean arterial pressure after aerobic exercise

In total, 55 independent comparisons were included in the SBP analysis. Meta-analysis demonstrated a *small* significant decrease in SBP after AE (SMD = -0.35; 95% CI -0.52 to -0.18; p = 0.0001; I^2^ = 70.15%). A similar effect was observed when the fixed-effect model was used (SMD = -0.26; 95% CI -0.36 to -0.18; p = 0.0001; I^2^ = 71.56%). Following the exclusion of one study [5], the random-effects model produced a similar magnitude of effect (SMD = -0.32; 95% CI -0.49 to -0.16; p = 0.0001; I^2^ = 68.06%), supporting the robustness of the overall effect estimate. To facilitate clinical interpretation, the corresponding pooled effect, expressed as a mean difference was -3.94 mmHg and is presented in the Supplementary Table 6.

## Fig. 5 Changes in systolic blood pressure after aerobic exercise

In total, 53 independent comparisons were included in the DBP analysis. Meta-analysis demonstrated a *small* significant decrease in DBP after AE (SMD = -0.41; 95% CI -0.61 to -0.21; p = 0.0001; I^2^ = 75.64%). A similar effect was observed when the fixed-effect model was used (SMD = -0.35; 95% CI -0.44 to -0.25; p = 0.0001; I^2^ = 71.56%). Following the exclusion of one study [6], the random-effects model produced a similar magnitude of effect (SMD = -0.37; 95% CI -0.54 to -0.19; p = 0.0001; I^2^ = 70.09%), supporting the robustness of the overall effect estimate. To facilitate clinical interpretation, the corresponding pooled effect, expressed as a mean difference was -2.55 mmHg and is presented in the Supplementary Table 6.

## Fig. 6 Changes in diastolic blood pressure after aerobic exercise

***Effect of aerobic exercise on endothelial blood markers***

In total, 7 independent comparisons were included in the NO analysis. Meta-analysis demonstrated a *large* significant increase in NO after AE (SMD = 1.23; 95% CI 0.56 to 1.89; p = 0.0003; I^2^ = 76.71%). A *moderate* effect was observed when the fixed-effect model was used (SMD = 1.08; 95% CI 0.76 to 1.39; p = 0.0001; I^2^ = 73.92%). Following the exclusion of one study [7], the random-effects model produced a *moderate* effect (SMD = 0.97; 95% CI 0.46 to 1.47; p = 0.0002; I^2^ = 56.87%). These discrepancies between models highlight the substantial heterogeneity and emphasize the need for cautious interpretation. To facilitate clinical interpretation, the corresponding pooled effect, expressed as a mean difference was 6 µmol/L and is presented in the Supplementary Table 6.

## Fig. 7 Changes in nitric oxide after aerobic exercise

In total, 6 independent comparisons were included in the NO_x_ analysis. Meta-analysis demonstrated a *large* significant increase in NO_x_ after AE (SMD = 1.23; 95% CI 0.75 to 1.70; p = 0.0001; I^2^ = 51.09%). A similar effect was observed when the fixed-effect model was used (SMD = 1.22; 95% CI 0.89 to 1.56; p = 0.0001; I^2^ = 51.05%). Following the exclusion of one study [8], the random-effects model produced a *moderate* effect (SMD = 1.09; 95% CI 0.61 to 1.57; p = 0.0001; I^2^ = 42.58%). These discrepancies following exclusion of one study highlight the substantial heterogeneity and emphasize the need for cautious interpretation. To facilitate clinical interpretation, the corresponding pooled effect, expressed as a mean difference was 4.05 µmol/L and is presented in the Supplementary Table 6.

## Fig. 8 Changes in nitrogen oxides after aerobic exercise

In total, 7 independent comparisons were included in the ET-1 analysis. Meta-analysis demonstrated a *moderate* significant decrease in ET-1 after AE (SMD = -0.99; 95% CI -1.49 to -0.48; p = 0.0001; I^2^ = 58.96%). A similar effect was observed when the fixed-effect model was used (SMD = -0.94; 95% CI -1.27 to -0.62; p = 0.0001; I^2^ = 59.00%). Following the exclusion of one study [9], the random-effects model produced a similar magnitude of effect (SMD = -0.79; 95% CI -1.20 to -0.38; p = 0.0002; I^2^ = 30.75%), supporting the robustness of the overall effect estimate. To facilitate clinical interpretation, the corresponding pooled effect, expressed as a mean difference was -0.55 pg/mL and is presented in the Supplementary Table 6.

## Fig. 9 Changes in endothelin-1 after aerobic exercise

***Effect of aerobic exercise on cardiorespiratory fitness***

In total, 54 independent comparisons were included in the VO_2max_ analysis. Meta-analysis demonstrated a *moderate* significant increase in VO_2max_ after AE (SMD = 0.81; 95% CI 0.64 to 0.97; p = 0.0001; I^2^ = 65.10%). A similar effect was observed when the fixed-effect model was used (SMD = 0.74; 95% CI 0.64 to 0.83; p = 0.0001; I^2^ = 65.88%). Following the exclusion of one study [10], the random-effects model produced a similar magnitude of effect (SMD = 0.78; 95% CI 0.62 to 0.93; p = 0.0001; I^2^ = 60.75%), supporting the robustness of the overall effect estimate. To facilitate clinical interpretation, the corresponding pooled effect, expressed as a mean difference was 3.06 ml/kg/min and is presented in the Supplementary Table 6.

## Fig. 10 Changes in VO_2max_ after aerobic exercise

In total, 10 independent comparisons were included in the RER analysis. Meta-analysis demonstrated a *trivial,* non-significant increase in RER after AE (SMD = 0.01; 95% CI -0.16 to 0.18; p = 0.9229; I^2^ = 0.0%). An identical effect was observed when the fixed-effect model was used (SMD = 0.01; 95% CI -0.16 to 0.18; p = 0.9229; I^2^ = 0.0%). Following the exclusion of one study [11], the random-effects model produced a similar magnitude of effect (SMD = 0.01; 95% CI -0.18 to 0.16; p = 0.9225; I^2^ = 0.0%), supporting the robustness of the overall effect estimate. To facilitate clinical interpretation, the corresponding pooled effect, expressed as a mean difference was 0.002 ml/kg/min and is presented in the Supplementary Table 6.

## Fig. 11 Changes in respiratory exchange ratio after aerobic exercise

In total, 7 independent comparisons were included in the GET analysis. Meta-analysis demonstrated a *moderate,* non-significant increase in GET after AT (SMD = 0.85; 95% CI -0.19 to 1.89; p = 0.1116; I^2^ = 93.28%). In contrast, the fixed-effect model yielded a *moderate* significant effect (SMD = 0.62; 95% CI 0.37 to 0.87; p = 0.0001; I^2^ = 81.84%). Following the exclusion of one study [10], the random-effects model produced a *small* but significant effect (SMD = 0.43; 95% CI 0.02 to 0.84; p = 0.0382; I^2^ = 53.64%). These discrepancies between models highlight the substantial heterogeneity and emphasize the need for cautious interpretation. To facilitate clinical interpretation, the corresponding pooled effect, expressed as a mean difference was 1.61 ml/kg/min and is presented in the Supplementary Table 6.

## Fig. 12 Changes in gas-exchange threshold after aerobic exercise

***Effects of aerobic exercise on body composition***

In total, 40 independent comparisons were included in the BW analysis. Meta-analysis demonstrated a *trivial,* significant decrease in BW after AE (SMD = -0.13; 95% CI -0.24 to -0.02; p = 0.0241; I^2^ = 0.0%). An identical effect was observed when the fixed-effect model was used (SMD = -0.13; 95% CI -0.24 to -0.02; p = 0.0241; I^2^ = 0.0%). Following the exclusion of one study [12], the random-effects model produced a similareffect (SMD = -0.13; 95% CI -0.24 to -0.02; p = 0.0247; I^2^ = 0.0%). . To facilitate clinical interpretation, the corresponding pooled effect, expressed as a mean difference was -1.36 kg and is presented in the Supplementary Table 6.

## Fig. 13 Changes in body weight after aerobic exercise

In total, 11 independent comparisons were included in the BFM analysis. Meta-analysis demonstrated a *trivial,* significant decrease in BFM after AE (SMD = -0.24; 95% CI -0.44 to 0.03; p = 0.0241; I^2^ = 0.0%). An identical effect was observed when the fixed-effect model was used (SMD = -0.24; 95% CI -0.44 to -0.03; p = 0.0241; I^2^ = 0.0%). Following the exclusion of one study [6], the random-effects model produced a similar, but non-significat magnitude of effect (SMD = -0.18; 95% CI -0.39 to 0.003; p = 0.0996; I^2^ = 0.0%).. To facilitate clinical interpretation, the corresponding pooled effect, expressed as a mean difference was -1.76 kg and is presented in the Supplementary Table 6.

## Fig. 14 Changes in body fat mass after aerobic exercise

In total, 19 independent comparisons were included in the BF% analysis. Meta-analysis demonstrated a *small* significant decrease in BF% after AE (SMD = -0.36; 95% CI -0.56 to -0.17; p = 0.0003; I^2^ = 27.69%). A similar effect was observed when the fixed-effect model was used (SMD = -0.32; 95% CI -0.48 to -0.16; p = 0.0001; I^2^ = 33.61%). Following the exclusion of one study [6], the random-effects model produced a similar magnitude of effect (SMD = -0.29; 95% CI -0.45 to -0.12; p = 0.0008; I^2^ = 5.21%), supporting the robustness of the overall effect estimate. To facilitate clinical interpretation, the corresponding pooled effect, expressed as a mean difference was -2.14% and is presented in the Supplementary Table 6.

## Fig. 15 Changes in body fat percentage after aerobic exercise

In total, 49 independent comparisons were included in the BMI analysis. Meta-analysis demonstrated a *trivial* significant decrease in BMI after AE (SMD = -0.19; 95% CI -0.28 to -0.10; p = 0.0001; I^2^ = 0.0%). An identical effect was observed when the fixed-effect model was used (SMD = -0.19; 95% CI -0.28 to -0.10; p = 0.0001; I^2^ = 0.0%). Following the exclusion of one study [13], the random-effects model produced a similar magnitude of effect (SMD = -0.17; 95% CI -0.27 to -0.08; p = 0.0005; I^2^ = 0.0%), supporting the robustness of the overall effect estimate. To facilitate clinical interpretation, the corresponding pooled effect, expressed as a mean difference was -0.72 kg/m² and is presented in the Supplementary Table 6.

## Fig. 16 Changes in body mass index after aerobic exercise

In total, 10 independent comparisons were included in the SMM analysis. Meta-analysis demonstrated a *trivial,* non-significant increase in SMM after AE (SMD = 0.12; 95% CI -0.12 to 0.35; p = 0.3387; I^2^ = 0.0%). A similar effect was observed when the fixed-effect model was used (SMD = 0.12; 95% CI -0.12 to 0.35; p = 0.3387; I^2^ = 0.0%). Following the exclusion of one study [14], the random-effects model produced similar magnitude of effect (SMD = 0.04; 95% CI -0.20 to 0.29; p = 0.7313; I^2^ = 0.0%). These discrepancies following exclusion of one study highlight the substantial heterogeneity and emphasize the need for cautious interpretation. To facilitate clinical interpretation, the corresponding pooled effect, expressed as a mean difference was 0.96 kg and is presented in the Supplementary Table 6.

## Fig. 17 Changes in skeletal muscle mass after aerobic exercise

***Effects of aerobic exercise on blood lipid profile***

In total, 43 independent comparisons were included in the HDL analysis. Meta-analysis demonstrated a *small* significant increase in HDL after AE (SMD = 0.31; 95% CI 0.11 to 0.52; p = 0.0030; I^2^ = 74.63%). A similar effect was observed when the fixed-effect model was used (SMD = 0.30; 95% CI 0.20 to 0.40; p = 0.0001; I^2^ = 73.87%). Following the exclusion of one study [15], the random-effects model produced a similar magnitude of effect (SMD = 0.22; 95% CI 0.10 to 0.34; p = 0.0003; I^2^ = 22.07%), supporting the robustness of the overall effect estimate. To facilitate clinical interpretation, the corresponding pooled effect, expressed as a mean difference was 0.07 mmol/L and is presented in the Supplementary Table 6.

## Fig. 18 Changes in high-density lipoprotein after aerobic exercise

In total, 39 independent comparisons were included in the LDL analysis. Meta-analysis demonstrated a *small* significant decrease in LDL after AE (SMD = -0.23; 95% CI -0.33 to -0.12; p = 0.0001; I^2^ = 0.0%). An identical effect was observed when the fixed-effect model was used (SMD = -0.23; 95% CI -0.33 to -0.12; p = 0.0001; I^2^ = 0.0%). Following the exclusion of one study [13], the random-effects model produced a similar magnitude of effect (SMD = -0.21; 95% CI -0.2 to -0.10; p = 0.0001; I^2^ = 0.0%), supporting the robustness of the overall effect estimate. To facilitate clinical interpretation, the corresponding pooled effect, expressed as a mean difference was -0.17 mmol/L and is presented in the Supplementary Table 6.

## Fig. 19 Changes in low-density lipoprotein after aerobic exercise

In total, 38 independent comparisons were included in the TG analysis. Meta-analysis demonstrated a *small* significant decrease in TG after AE (SMD = -0.19; 95% CI -0.32 to -0.07; p = 0.0027; I^2^ = 22.20%). A similar effect was observed when the fixed-effect model was used (SMD = -0.18; 95% CI -0.29 to -0.07; p = 0.0012; I^2^ = 23.15%). Following the exclusion of one study [8], the random-effects model produced a similar magnitude of effect (SMD = -0.16; 95% CI -0.27 to -0.41; p = 0.0066; I^2^ = 4.56%), supporting the robustness of the overall effect estimate. To facilitate clinical interpretation, the corresponding pooled effect, expressed as a mean difference was -0.08 mmol/L and is presented in the Supplementary Table 6.

## Fig. 20 Changes in triglyceride after aerobic exercise

In total, 40 independent comparisons were included in the tCHO analysis. Meta-analysis demonstrated a *trivial,* non-significant increase in tCHO after AE (SMD = 0.02; 95% CI -0.20 to 0.24; p = 0.8431; I^2^ = 76.82%). A similar effect was observed when the fixed-effect model was used (SMD = 0.08; 95% CI -0.03 to 0.18; p = 0.1427; I^2^ = 80.57%). Following the exclusion of one study [15], the random-effects model produced a similar magnitude of effect (SMD = -0.05; 95% CI -0.23 to 0.13; p = 0.5751; I^2^ = 60.88%), supporting the robustness of the overall effect estimate. To facilitate clinical interpretation, the corresponding pooled effect, expressed as a mean difference was -0.05 mmol/L and is presented in the Supplementary Table 6.

## Fig. 21 Changes in total cholesterol after aerobic exercise

***Effect of aerobic exercise on glucose metabolism markers***

In total, 28 independent comparisons were included in the glucose analysis. Meta-analysis demonstrated a *small* significant decrease in glucose after AE (SMD = -0.27; 95% CI -0.43 to -0.11; p = 0.0008; I^2^ = 27.81%). A similar effect was observed when the fixed-effect model was used (SMD = -0.25; 95% CI -0.38 to -0.12; p = 0.0001; I^2^ = 28.62%). Following the exclusion of one study [16], the random-effects model produced a similar magnitude of effect (SMD = -0.23; 95% CI -0.38 to -0.09; p = 0.0017; I^2^ = 15.23%), supporting the robustness of the overall effect estimate. To facilitate clinical interpretation, the corresponding pooled effect, expressed as a mean difference was -0.23 mmol/L and is presented in the Supplementary Table 6.

## Fig. 22 Changes in glucose after aerobic exercise

In total, 16 independent comparisons were included in the insulin analysis. Meta-analysis demonstrated a *trivial,* non-significant decrease in insulin after AE (SMD = -0.19; 95% CI -0.67 to 0.28; p = 0.4294; I^2^ = 87.04%). A similar effect, but significant was observed when the fixed-effect model was used (SMD = -0.19; 95% CI -0.35 to 0.02; p = 0.0246; I^2^ = 80.29%). Following the exclusion of one study [17], the random-effects model produced a similar magnitude of effect (SMD = -0.11; 95% CI -0.59 to 0.36; p = 0.6462; I^2^ = 86.46%). These discrepancies between models highlight the substantial heterogeneity and emphasize the need for cautious interpretation. To facilitate clinical interpretation, the corresponding pooled effect, expressed as a mean difference was 0.03 µIU/mL and is presented in the Supplementary Table 6.

## Fig. 23 Changes in insulin after aerobic exercise

In total, 11 independent comparisons were included in the HbA1c analysis. Meta-analysis demonstrated a *small* significant decrease in HbA1c after AE (SMD = -0.35; 95% CI -0.63 to -0.07; p = 0.0149; I^2^ = 28.97%). A similar effect was observed when the fixed-effect model was used (SMD = -0.35; 95% CI -0.58 to -0.11; p = 0.0036; I^2^ = 28.11%). Following the exclusion of one study [8], the random-effects model produced a similar magnitude of effect (SMD = -0.25; 95% CI -0.49 to -0.01; p = 0.0449; I^2^ = 0.0%), supporting the robustness of the overall effect estimate. To facilitate clinical interpretation, the corresponding pooled effect, expressed as a mean difference was -0.35% and is presented in the Supplementary Table 6.

## Fig. 24 Changes in glycated haemoglobin after aerobic exercise

In total, 16 independent comparisons were included in the HOMA-IR analysis. Meta-analysis demonstrated a *small* significant decrease in HOMA-IR after AE (SMD = -0.54; 95% CI -0.83 to -0.25; p = 0.0003; I^2^ = 64.01%). A similar effect was observed when the fixed-effect model was used (SMD = -0.40; 95% CI -0.56 to -0.23; p = 0.0001; I^2^ = 63.66%). Following the exclusion of one study [16], the random-effects model produced a similar magnitude of effect (SMD = -0.49; 95% CI -0.77 to -0.20; p = 0.0009; I^2^ = 62.10%), supporting the robustness of the overall effect estimate. To facilitate clinical interpretation, the corresponding pooled effect, expressed as a mean difference was -0.73% and is presented in the Supplementary Table 6.

## Fig. 25 Changes in homeostatic model assessment for insulin resistance after aerobic exercise

***Effect of aerobic exercise on inflammation markers***

In total, 20 independent comparisons were included in the CRP analysis. Meta-analysis demonstrated a *small* significant decrease in CRP after AE (SMD = -0.36; 95% CI -0.61 to -0.11; p = 0.0049; I^2^ = 54.55%). A similar effect was observed when the fixed-effect model was used (SMD = -0.31; 95% CI -0.48 to -0.15; p = 0.0002; I^2^ = 54.96%). Following the exclusion of one study [18], the random-effects model produced a similar magnitude of effect (SMD = -0.29; 95% CI -0.50 to -0.08; p = 0.0073; I^2^ = 34.38%), supporting the robustness of the overall effect estimate. To facilitate clinical interpretation, the corresponding pooled effect, expressed as a mean difference was -2.12 mg/L and is presented in the Supplementary Table 6.

## Fig. 26 Changes in C-reactive protein after aerobic exercise

In total, 7 independent comparisons were included in the TNF-α analysis. Meta-analysis demonstrated a *small,* non-significant decrease in TNF-α after AE (SMD = -0.39; 95% CI -0.87 to 0.10; p = 0.1186; I^2^ = 60.98%). A similar effect, but significant, was observed when the fixed-effect model was used (SMD = -0.32; 95% CI -0.61 to -0.02; p = 0.0375; I^2^ = 58.95%). Following the exclusion of one study [18], the random-effects model produced a *trivial* magnitude of effect (SMD = -0.17; 95% CI -0.48 to 0.13; p = 0.2682; I^2^ = 0.0%). These discrepancies between models highlight the substantial heterogeneity and emphasize the need for cautious interpretation. To facilitate clinical interpretation, the corresponding pooled effect, expressed as a mean difference was –0.72 pg/mL and is presented in the Supplementary Table 6.

## Fig. 27 Changes in tumor necrotic factor-α after aerobic exercise

In total, 7 independent comparisons were included in the IL-6 analysis. Meta-analysis demonstrated a *trivial,* non-significant decrease in IL-6 after AE (SMD = -0.17; 95% CI -0.48 to 0.15; p = 0.2996; I^2^ = 13.61%). A similar effect was observed when the fixed-effect model was used (SMD = -0.17; 95% CI -0.46 to 0.13; p = 0.2667; I^2^ = 9.90%). Following the exclusion of one study [9], the random-effects model produced a similar magnitude of effect (SMD = -0.07; 95% CI -0.38 to 0.25; p = 0.6827; I^2^ = 0.0%), supporting the robustness of the overall effect estimate. To facilitate clinical interpretation, the corresponding pooled effect, expressed as a mean difference was –0.19 pg/mL and is presented in the Supplementary Table 6.

## Fig. 28 Changes in interleukin 6 after aerobic exercise

# References

1. Aispuru-Lanche R, Jayo-Montoya JA, Maldonado-Martín S. Vascular-endothelial adaptations following low and high volumes of high-intensity interval training in patients after myocardial infarction. Ther Adv Cardiovasc Dis. SAGE Publications Ltd; 2024;18. https://doi.org/10.1177/17539447241286036

2. Shenouda N, Gillen JB, Gibala MJ, MacDonald MJ. Changes in brachial artery endothelial function and resting diameter with moderate-intensity continuous but not sprint interval training in sedentary men. J Appl Physiol. 2017;123:773–80. https://doi.org/10.1152/japplphysiol.00058.2017

3. Huang J, Leng L, Hu M, Cui X, Yan X, Liu Z, et al. Comparative Effects of Different Exercise Types on Cardiovascular Health and Executive Function in Sedentary Young Individuals. Med Sci Sports Exerc. 2025;57:1110–22. https://doi.org/10.1249/MSS.0000000000003645

4. Kirkman DL, Ramick MG, Muth BJ, Stock JM, Pohlig RT, Townsend RR, et al. Effects of aerobic exercise on vascular function in nondialysis chronic kidney disease: a randomized controlled trial. American Journal of Physiology-Renal Physiology. 2019;316:F898–905. https://doi.org/10.1152/ajprenal.00539.2018

5. Beck DT, Casey DP, Martin JS, Emerson BD, Braith RW. Exercise training improves endothelial function in young prehypertensives. Exp Biol Med. 2013;238:433–41. https://doi.org/10.1177/1535370213477600

6. Azadpour N, Tartibian B, Koşar ŞN. Effects of aerobic exercise training on ACE and ADRB2 gene expression, plasma angiotensin II level, and flow-mediated dilation: a study on obese postmenopausal women with prehypertension. Menopause. 2017;24:269–77. https://doi.org/10.1097/GME.0000000000000762

7. He H, Wang C, Chen X, Sun X, Wang Y, Yang J, et al. The effects of HIIT compared to MICT on endothelial function and hemodynamics in postmenopausal females. J Sci Med Sport. 2022;25:364–71. https://doi.org/10.1016/j.jsams.2022.01.007

8. Abdi S, Tadibi V, Sheikholeslami-Vatani D. Effect of High-intensity Interval Training on Endothelial Function in Type 2 Diabetic Females. Asian J Sports Med. 2021;12. https://doi.org/10.5812/asjsm.113566

9. Kobayashi N, Tsuruya Y, Iwasawa T, Ikeda N, Hashimoto S, Yasu T, et al. Exercise Training in Patients With Chronic Heart Failure Improves Endothelial Function Predominantly in the Trained Extremities. Circulation Journal. 2003;67:505–10. https://doi.org/10.1253/circj.67.505

10. Eleuteri E, Mezzani A, Di Stefano A, Vallese D, Gnemmi I, Delle Donne L, et al. Aerobic training and angiogenesis activation in patients with stable chronic heart failure: a preliminary report. Biomarkers. 2013;18:418–24. https://doi.org/10.3109/1354750X.2013.805342

11. Wisløff U, Støylen A, Loennechen JP, Bruvold M, Rognmo Ø, Haram PM, et al. Superior cardiovascular effect of aerobic interval training versus moderate continuous training in heart failure patients: A randomized study. Circulation [Internet]. Lippincott Williams & Wilkins; 2007 [cited 2024 Aug 22];115:3086–94. https://doi.org/10.1161/CIRCULATIONAHA.106.675041/ASSET/6947D37C-D1BB-4ECE-AF16-0BED1308C093/ASSETS/GRAPHIC/12FF2.JPEG

12. Ghardashi Afousi A, Izadi MR, Rakhshan K, Mafi F, Biglari S, Gandomkar Bagheri H. Improved brachial artery shear patterns and increased flow‐mediated dilatation after low‐volume high‐intensity interval training in type 2 diabetes. Exp Physiol. 2018;103:1264–76. https://doi.org/10.1113/EP087005

13. Davoodi M, Hesamabadi BK, Ariabood E, Izadi MR, Ghardashi‐Afousi A, Bigi MAB, et al. Improved blood pressure and flow‐mediated dilatation via increased plasma adropin and nitrate/nitrite induced by high‐intensity interval training in patients with type 2 diabetes. Exp Physiol. 2022;107:813–24. https://doi.org/10.1113/EP089371

14. Eaungpulsawat W, Suksom D. Effectiveness of the Modified Otago Exercise Program on Blood Pressure and Vascular Function in Older Adults with Hypertension. J Exerc Physiol Online [Internet]. 2023;26:51–9. https://research.ebsco.com/linkprocessor/plink?id=03566fb3-aa2b-3dd8-90f1-c72c00647715

15. Sherwood A, Blumenthal JA, Smith PJ, Watkins LL, Hoffman BM, Hinderliter AL. Effects of Exercise and Sertraline on Measures of Coronary Heart Disease Risk in Patients With Major Depression: Results From the SMILE-II Randomized Clinical Trial. Psychosom Med. 2016;78:602–9. https://doi.org/10.1097/PSY.0000000000000301

16. Tjønna AE, Lee SJ, Rognmo Ø, Stølen TO, Bye A, Haram PM, et al. Aerobic Interval Training Versus Continuous Moderate Exercise as a Treatment for the Metabolic Syndrome. Circulation. 2008;118:346–54. https://doi.org/10.1161/CIRCULATIONAHA.108.772822

17. Desch S, Sonnabend M, Niebauer J, Sixt S, Sareban M, Eitel I, et al. Effects of physical exercise versus rosiglitazone on endothelial function in coronary artery disease patients with prediabetes. Diabetes Obes Metab. 2010;12:825–8. https://doi.org/10.1111/j.1463-1326.2010.01234.x

18. Robinson AT, Franklin NC, Norkeviciute E, Bian JT, Babana JC, Szczurek MR, et al. Improved arterial flow-mediated dilation after exertion involves hydrogen peroxide in overweight and obese adults following aerobic exercise training. [Internet]. J. Hypertens. Netherlands: Wolters Kluwer Health, Inc; 2016. p. 1309–16. https://doi.org/10.1097/HJH.0000000000000946
